# Gene Expression Correlation Analysis Reveals MYC-NAC Regulatory Network in Cotton Pigment Gland Development

**DOI:** 10.3390/ijms22095007

**Published:** 2021-05-08

**Authors:** Hailiang Cheng, Xiaoxu Feng, Dongyun Zuo, Youping Zhang, Qiaolian Wang, Limin Lv, Chaofeng Wu, Shuyan Li, Yuanli Dai, Da Qu, Man He, Shang Liu, Guoli Song

**Affiliations:** 1 State Key Laboratory of Cotton Biology, Institute of Cotton Research of the Chinese Academy of Agricultural Sciences, Anyang 455000, China; pser2010@163.com (H.C.); bbxe2013@163.com (X.F.); zdy041@163.com (D.Z.); zyp547550790@163.com (Y.Z.); wangqiaolian@caas.cn (Q.W.); llm0372@126.com (L.L.); wuchaofeng7811@163.com (C.W.); lishuyan6688@163.com (S.L.); daiyuanli1997@163.com (Y.D.); qd_neau@163.com (D.Q.); manhe199641@163.com (M.H.); lsxbdz@163.com (S.L.); 2 Plant Genetics, Gembloux Agro Bio-Tech, University of Liège, 5030 Gembloux, Belgium

**Keywords:** NAC transcription factors, GhMYC2-like, cotton pigment gland, regulatory network

## Abstract

Plant NAC (NAM, ATAF1/2, and CUC2) family is involved in various development processes including Programmed Cell Death (PCD) associated development. However, the relationship between NAC family and PCD-associated cotton pigment gland development is largely unknown. In this study, we identified 150, 153 and 299 *NAC* genes in newly updated genome sequences of *G. arboreum*, *G. raimondii* and *G. hirsutum*, respectively. All *NAC* genes were divided into 8 groups by the phylogenetic analysis and most of them were conserved during cotton evolution. Using the vital regulator of gland formation *GhMYC2-like* as bait, expression correlation analysis screened out 6 *NAC* genes which were low-expressed in glandless cotton and high-expressed in glanded cotton. These 6 *NAC* genes acted downstream of *GhMYC2-like* and were induced by MeJA. Silencing *CGF1* *(Cotton Gland Formation1)*, another MYC-coding gene, caused almost glandless phenotype and down-regulated expression of *GhMYC2-like* and the *6 NAC* genes, indicating a MYC-NAC regulatory network in gland development. In addition, predicted regulatory mechanism showed that the *6* *NAC* genes were possibly regulated by light, various phytohormones and transcription factors as well as miRNAs. The interaction network and DNA binding sites of the 6 NAC transcription factors were also predicted. These results laid the foundation for further study of gland-related genes and gland development regulatory network.

## 1. Introduction

Cotton is an important economic crop providing natural fiber resource and potential nutrition resource. For example, global cottonseed production, if being fully utilized, could provide sufficient protein consumption for 500 million people one year [1]. Nevertheless, presence of toxic gossypol in pigment gland limited the use of the cottonseed production due to its toxicity for human and monogastric animals [2,3]. Besides, gossypol is phytoalexin providing plant resistance to various insects and pathogens [4,5,6,7]. It is significant for improving cotton economic value to breed cotton cultivars with high gossypol in plant and low gossypol in seed. In 2006, Sunikumar et al. created low-gossypol cotton using RNAi technology to suppress specially the expression of δ-cadinene synthase genes in cottonseed, which are key factors in gossypol biosynthesis [8,9,10,11]. The transgenic cotton showed reduced gossypol content in seed and normal in foliage and floral parts [10,12].

Pigment glands or gossypol glands are the specific organ of *Gossypium* spp. The gland is a cavity structure formed by lysigenous intercellular space generated by programmed cell death (PCD) [13,14]. As the main storage of gossypol, mature glands contain plenty of gossypol and various terpenoid aldehydes [11,15]. Gossypol content was reported to be closely related to pigment gland number in cotton [16,17,18]. However, compared with gossypol biosynthesis, little is known about the mechanism underlying pigment gland organogenesis. Up to now, six independent gene loci, named *Gl_1_*, *Gl_2_*, *Gl_3_*, *Gl_4_*, *Gl_5_* and *Gl_6_*, were identified to be involved in gland formation. The first reported one is recessive gene *gl_1_*, which controlling phenotype of glanded cotyledon and leaf, and glandless hypocotyls, petiole, boll shell and stem [19]. Then, homozygous recessive *gl_2_gl_2_gl_3_gl_3_* were reported to confer completely glandless phenotype of upland cotton plant [20,21]. Allele *gl_4_* and *gl_5_* contribute to reducing gland density and *gl_6_* is similar to *gl_1_* with weaker function [22,23]. One dominant gene *Gl_2_^e^* is allele of *Gl_2_* and responsible for whole-plant glandless phenotype of mutant ‘Bahtim 110′ from radiation mutagenesis of ‘Giza45′ (*G. barbadense* L.) [24,25]. Since then, many efforts were made to isolate the genes relate to gland formation. In 2016, *Gl_2_^e^* was mapped to a 15 kb region on chromosome A12 and a basic helix-loop-helix (bHLH) transcription factor GhMYC2-like was identified and supposed to be involved in gland formation because of an amino acid substitution in conserved domain and low expression level in glandless cotton [26]. The transcription factor was functionally confirmed by virus induced gene silencing (VIGS) and named *Gossypium Pigment Gland Formation* (*GoPGF*). The gene on chromosome A12 and D12 were identified as *Gl_2_* and *Gl_3_*, and *gl_2_* and *gl_3_* were revealed to be derived from the premature truncated *Gl_2_* and *Gl_3_*, respectively [27]. Then, three *Cotton Gland Formation* (*CGF*) genes were identified by transcriptome analysis of glanded and glandless cotton embryos. Silencing *CGF1* and *CGF3* (synonym *GoPGF*) dramatically suppressed gland formation and the critical role of *CGF3* in gland formation was further confirmed by CRISPR/Cas9-mediated knockout of the gene. *CGF2* encoding NAC transcription factors have a mild regulating effect on gland formation [28]. Although many efforts were made recently [29,30], the molecular mechanism of gland development was still unclear.

NAC (NAM, ATAF1/2, CUC2) transcription factors are plant-specific proteins and belong to one of the largest transcription factor families [31]. There are a highly conserved DNA-binding domain and a variable transcriptional regulatory region at N-terminal and C-terminal of NAC transcription factors, respectively [32]. The first *NAC* gene was identified in *Petunia*, which controlling development of shoot apical meristem (SAM) [33]. Then, *NAC* genes were identified in various plants, including *Arabidopsis thaliana* [34], *Oryza sativa* [35], *Vitis vinifera* [36] and *Gossypium* spp. [37,38,39,40,41]. Increasing evidences indicate NACs involved in plant abiotic and biotic response and development regulation [42,43,44,45,46]. It is worth mentioning that NACs participate in PCD-associated vegetative development [47]. For example, a XYLEM NAC DOMAIN 1 (XND1) negatively regulated lignocellulose synthesis and PCD in xylem [48], VASCULAR-RELATED NAC DOMAIN 6 (VND6) and VND7 can activate PCD in xylem vessel element differentiation and VND7 directly bind the promoter of XND1 to regulate its expression [49,50]. In leaf senescence process, NAC transcription factor ORE1 directly targets senescence-induced BIFUNCTIONAL NUCLEASE1 (BFN1) to regulate PCD in Arabidopsis [51]. ANAC033/SOMBRERO controls the PCD in lateral root cap differentiation [52]. Recently, ANAC087 and ANAC046 were also reported to regulate PCD in the columella and lateral root cap [53].

Gland formation is a PCD-associated development process and one pair of *NAC* genes (*CGF2*) are reported to affect gland density. *NAC* genes form one large gene family and previous studies focus on functions of NAC family members in stress responses and fiber development in cotton [37,38,39,40,41]. It is unknown whether other *NAC* genes participate in gland development regulation. In this study, genome-wide identification and expression correlation analysis of *NAC* genes in cotton were performed and 6 *NAC* genes were screened out to be supposed to involve in gland formation. Further analysis revealed that they acted downstream of *GhMYC2-like* and were induced by MeJA. Besides, Another MYC transcription factor CGF1 influenced gland formation by regulating *GhMYC2-like* and these 6 *NAC* genes. These results delineated a MYC-NAC regulatory network in gland development. In addition, the regulatory mechanism of 6 *NAC* genes was predicted. This study laid the foundation for further analysis of gland-related *NAC* genes and gland development regulatory network.

## 2. Results

### 2.1. Identification and Phylogenetic Analysis of NAC Genes Family in Cotton

To identify the *NAC* genes, recently updated genome reference sequences of *gossypium* spp. were downloaded from database (https://www.cottongen.org/, accessed on 20 June 2020). Total 299, 150 and 153 NAC proteins were obtained in *G. hirsutum*, *G. arboreum* and *G. raimondii* respectively using hmmsearch and blastp search. Then, the conserved domain in NAC proteins were confirmed using online software PfamScan (https://www.ebi.ac.uk/Tools/pfa/pfamscan/, accessed on 20 June 2020). The NAC proteins in 3 cotton genomes were named Gh_NAC1-Gh_NAC299, Ga_NAC1-Ga_NAC150 and Gr_NAC1-Gr_NAC153, respectively. The characteristics of NAC proteins were analyzed. For example, the range of length of NAC proteins were from 87 (Gh_NAC100) to 860 (Gh_NAC140) amino acids, 66 (Ga_NAC5) to 1133 (Ga_NAC18) amino acids and 123 (Gr_NAC131) to 859 (Gr_NAC100) amino acids. The range of isoelectric point of NAC proteins were from 4.102 (Gh_NAC160) to 10.281 (Gh_NAC111), 4.445 (Ga_NAC68) to 10.14 (Ga_NAC121) and 4.381 (Gr_NAC117) to 10.65 (Gr_NAC144). The grand average of hydropathy of NAC proteins were range from −1.001 (Gh_NAC259) to −0.187 (Gh_NAC189), −0.956 (Ga_NAC55) to −0.335 (Ga_NAC9) and −0.977 (Gr_NAC119) to −0.293 (Gr_NAC131). Subcellular localization prediction showed that about 3/4 NAC proteins located in nucleus and 1/4 were extracellular, except for Ga_NAC3 (membrane), Ga_NAC97 (chloroplast) and Gr_NAC69 (chloroplast). Detail information of each NAC protein was listed in Tables S2–S4.

**Figure 1:**
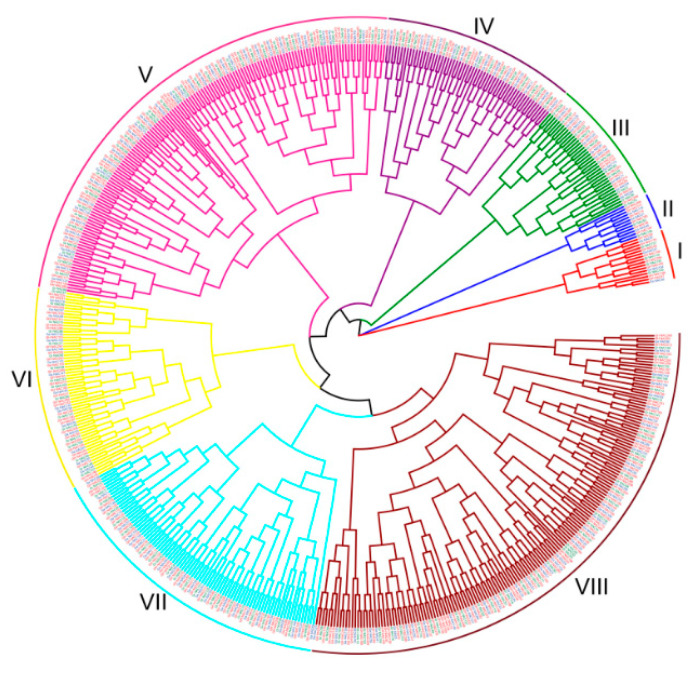
Phylogenetic analysis of NAC proteins from three cotton genomes. The labels in color indicate NAC proteins form *G. hirsutum* (red), *G. arboreum* (blue) and *G. raimondii* (green), respectively.

A maximum likehood (ML) tree was constructed to uncover the phylogenetic relationship of the NAC proteins in *G. hirsutum*, *G. arboreum* and *G. raimondii* (Figure 1). The 602 NAC proteins were divided into 8 groups (I–VIII). Almost all *NAC* genes in *G. arboreum* and *G. raimondii*, which are genome progenitors of *G. hirsutum*, have the orthologous genes in *G. hirsutum*. There were some exceptions, such as *Ga_NAC5*, *Ga_NAC19*, *Ga_NAC49*, *Ga_NAC62*, *Ga_NAC129*, *Gr_NAC1*, *Gr_NAC49*, *Gr_NAC55*, *Gr_NAC125*, *Gr_NAC131*, *Gr_NAC133* and *Gr_NAC146* without orthologs in *G. hirsutum*, which indicating the gene loss in allotetraploid or gene gain in diploid cotton. Gene gain was also observed during evolution of *G. hirsutum*, such as *Gh_NAC255/Gh_NAC256*, *Gh_NAC106/Gh_NAC107* and *Gh_NAC188/Gh_NAC189* share one ortholog in *G. raimondii*, respectively.

### 2.2. Gene Chromosomal Location, Duplication and Synteny Analysis of NACs in Cotton

Total 298 out of 299 *Gh_NACs* were distributed on all chromosomes with number range from 7 to 17 and the other one located on scaffold (Figure S1A,B). *Ga_NACs* and *Gr_NACs* distributed on corresponding chromosomes with similar pattern (Figure S1C,D). Gene duplication analysis revealed that 231, 18 and 10 duplicated gene pairs of *NAC* genes derived from segmental duplication in *G. hirsutum*, *G. arboreum* and *G. raimondii*, respectively (Table S5 and Figures S2–S4). Most *Gh_NAC* gene duplications were derived from allotetraploid formation (duplicated gene pairs from A and D subgenome). Several *NACs* had more than one duplicated genes implying their contribution to *NAC* gene family expansion. Meanwhile, tandem duplication of *NACs* were also detected (Table S5), such as *Gh_NAC85/Gh_NAC86/Gh_NAC87, Gh_100/Gh_NAC101, Ga_NAC25/Ga_NAC26, Ga_NAC83/Ga_NAC84/Ga_NAC85, Gr_NAC41/Gr_NAC42/Gr_NAC43, Gr_NAC54/Gr_NAC55.* Ka/Ks ratio of each pair of *NAC* genes was calculated to explore the selective pressure in evolution. As shown in Table S5, most duplicated *NAC* gene pairs had a Ka/Ks ratio < 1, which undergoing purifying selection. Seven pairs with Ka/Ks ratio > 1 were subjected to positive selection, including *Gh_NAC14/Gh_NAC164*, *Gh_NAC33/Gh_NAC200*, *Gh_NAC89/Gh_NAC239*, *Gh_NAC122/Gh_NAC271*, *Gh_NAC81/Gh_NAC231*, *Gh_NAC28/Gh_NAC170* and *Gh_NAC72/Gh_NAC222*.

**Figure 2:**
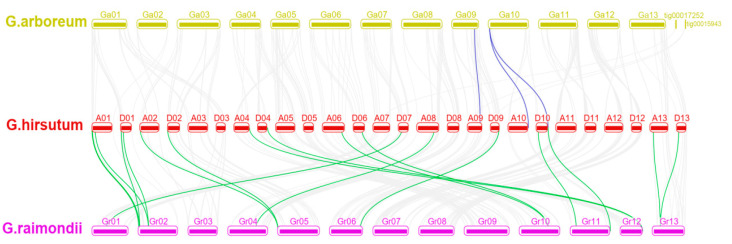
Synteny analysis of *NACs* in cotton. The grey lines indicate *NACs* collinear relationship in three genomes and color lines indicate that only in two genomes. Ga: *Gossypium arboreum*, Gr: *Gossypium raimondii*, A/D: A/D subgenome of *Gossypium hirsutum*. The numbers indicate chromosome number. Tig means contig.

**Figure 3:**
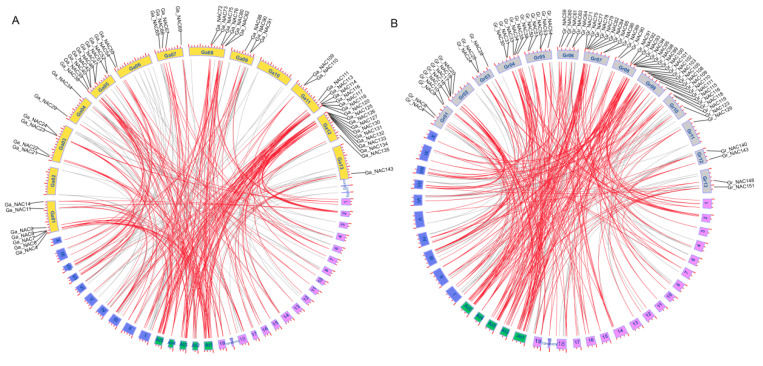
Synteny analysis of *NACs* between diploid cotton and Arabidopsis, cacao and grape. Blue, green, purple, yellow (**A**) and grey (**B**) blocks indicate the chromosomes of cacao, Arabidopsis, grape, *G. arboreum* (**A**) and *G. raimondii* (**B**), respectively. The gene labels and red lines indicate collinear relationship of genes with high conservation.

To investigate the synteny relationship of *NAC* genes, total 114, 100 and 74 *NAC* genes were identified in *Arabidopsis thaliana*, *Theobroma cacao* and *Vitis vinifera*. Synteny analysis showed that *NAC* genes had high collinear relationship in three cotton genomes, as well as between diploid cotton and *A. thaliana*, *T.* *cacao* and *V. vinifera*, respectively (Figure 2 and Figure 3 and Table S6). Total 115/124, 109/114 and 96/104 orthologous *NAC* gene pairs were detected between *G. arboreum*/*G. raimondii* and *A. thaliana*, *T.* *cacao* and *V. vinifera*, respectively (Table S7). Among them, orthologous genes of 55 *Ga_NACs* and 65 *Gr_NACs* were detected in all three plants suggesting that they were generated before the species divergence and highly conserved during species differentiation (Figure 3). Besides, we explored the occurrence time of *NACs* tandem duplication. Corresponding tandem duplications of *Ga_NAC114/Ga_NAC115*, *Gr_NAC80/Gr_NAC81*, *Gh_NAC121/Gh_NAC122* and *Gh_NAC270/Gh_NAC271* were observed in cacao indicating that the duplication occurred at 33 million years ago (mya), which is divergence time of cotton ancestor and cacao [54]. Another tandem duplication event *Ga_NAC49/Ga_NAC50* was detected in cacao but not in *G. raimondii* and *G. hirsutum*, suggesting that the duplication occurred at 33 mya and lost in *G. raimondii* and *G. hirsutum* after species differentiation. Some tandem duplications only existed in cotton implying that they were generated at 5–10 mya before *G. arboreum* and *G. raimondii* diverged from the common ancestor. These duplications were also retained in *G. hirsutum*, including *Ga_NAC83/Ga_NAC84/Ga_NAC85*, *Gr_NAC41/Gr_NAC42/Gr_NAC43*, *Ga_NAC97/Ga_NAC98* and *Gr_NAC69/Gr_NAC70* (Table S6). Other tandem duplications of *NAC* genes generated and evolved independently in each cotton species in recent 1–2 Mya after the allotetraploid formation. These results suggested *NAC* gene family experienced complex gene gain and loss during *Gossypium* evolution.

### 2.3. Identification of Gland Formation Associated NAC Genes in G. hirsutum

**Figure 4:**
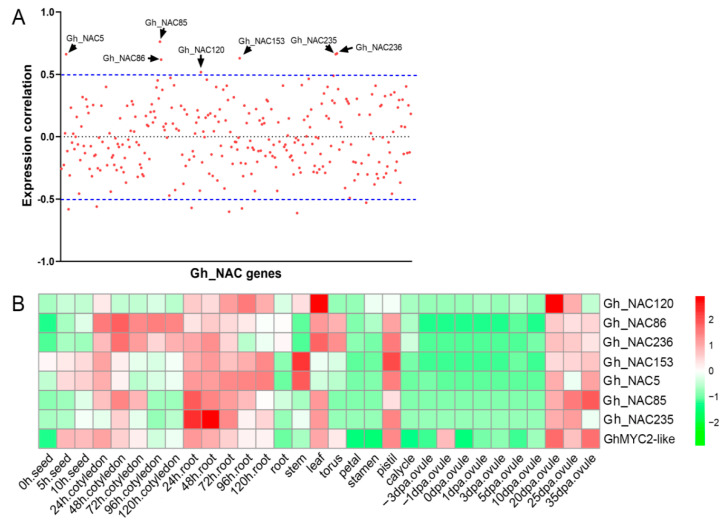
Expression correlation analysis of *GhMYC2-like* and *NAC* genes. (**A**) Distribution of expression correlation. The red dots indicate genes. Black arrows indicate the genes co-expressed with *GhMYC2-like* (**B**) Relative expression levels of *GhMYC2-like* and *7 NAC* genes in different tissues and development stages of TM-1. Dpa: Day Post Anthesis.

The transcription factor GhMYC2-like (synonym GoPGF) was identified as a vital regulator in pigment gland development [26,27,28]. To identify the *NAC* genes involved in pigment gland development, spatiotemporal expression analysis of all *Gh_NAC* genes and *GhMYC2-like* were conducted using the public RNA-seq data. Expression correlation of *GhMYC2-like* and each *NAC* gene was calculated and 7 *NAC* genes were identified as being co-expressed with *GhMYC2-like* (correlation > 0.5) (Figure 4A). Among of them, *Gh_NAC120* belonged to group V and other Six (3 pair of alleles including 2 duplicated gene pairs) were from group VII (Figure 1 and Table S5). Notably, as shown in Figure 4B, the 7 *NAC* genes showed low expression level in ovule before 10 dpa and high expression level after 20 dpa, which in line with gland development time that starting at 14–16 dpa [28]. The result suggested that these genes were likely to participate in gland development regulation.

**Figure 5:**
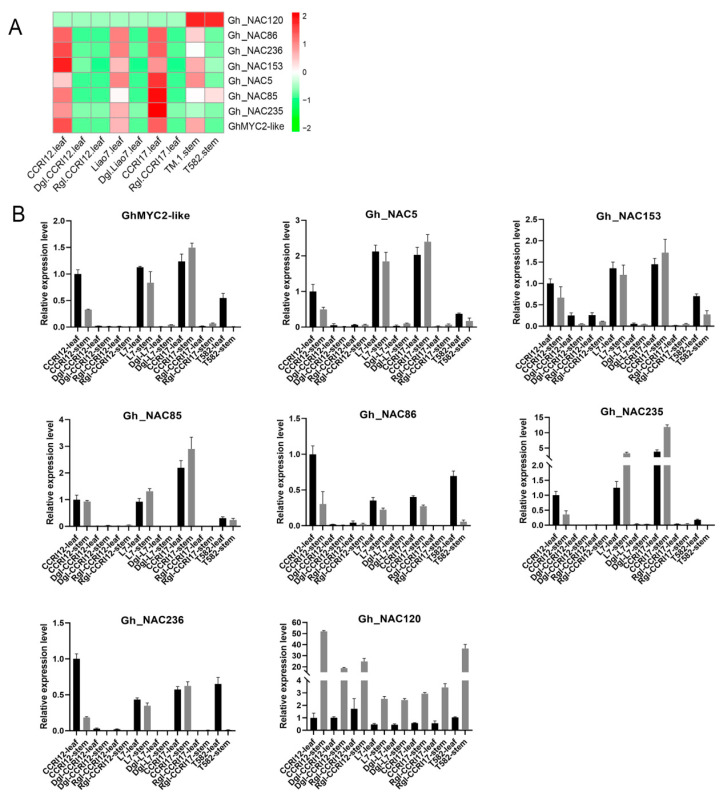
Expression analysis of *GhMYC2-like* and 7 *NAC* genes in glanded and glandless cotton (reference gene: actin). (**A**) Relative expression levels of *GhMYC2-like* and 7 *NAC* genes in glanded and glandless cotton. (**B**) QRT-PCR analysis of *GhMYC2-like* and 7 *NAC* genes in glanded and glandless cotton. CCRI: China Cotton Research Institute, Dgl: dominant glandless, Rgl: recessive glandless, L7: Liaomian7.

To confirm whether 7 *NAC* genes were relative to glandless phenotype, the genes expression were checked in glanded cotton CCRI12 (China Cotton Research Institute 12), Liao7, CCRI17, dominant glandless (Dgl) cotton Dgl-CCRI12, Dgl-Liao7 and recessive glandless (Rgl) cotton Rgl-CCRI12 and Rgl-CCRI17, as well as stem-glandless line T582. According to our RNA sequencing data, except for *Gh_NAC120*, the expression of other 6 *NAC* genes and *GhMYC2-like* were lower in leaves or stems of glandless cotton than that in glanded cotton (Figure 5A). Meanwhile, qRT-PCR was employed to verify the expression pattern of 7 *NAC* genes and *GhMYC2-like* in leaves and stems of all these cotton lines. Previous studies revealed stable reference gene expression is important for accurate quantification of gene expression [55,56]. In this study, we used 3 reference genes (actin, ubiquitin and histone) for qRT-PCR analysis. As shown in Figure 5B, *Gh_NAC120* was highly expressed in all stems of materials regardless of gland phenotype suggesting that it was not associated to gland formation. The other 6 *NAC* genes and *GhMYC2-like* were at low expression level in all glandless materials in accord with the RNA-seq results. There were similar results by using 3 reference genes as internal controls, suggesting the expression analysis was relatively accurate and each reference gene was available for qPCR analysis (Figure S5). Among of 6 genes, the function of *Gh_NAC5/Gh_NAC153* (assigned as *CGF2*) was verified in gland formation by gene knockout [28]. These results indicated *GhMYC2-like* probably functioned together with these 6 *NAC* genes in gland development.

### 2.4. The Gland-Related NACs Function Downstream of GhMYC2-Like

**Figure 6:**
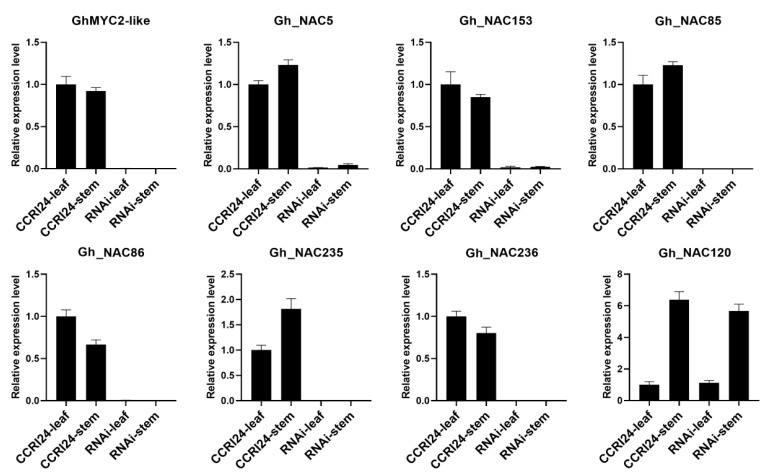
QRT-PCR analysis of *GhMYC2-like* and 7 *NAC* genes in CCRI24 and RNAi plants (reference gene: actin).

Normal expression of *GhMYC2-like* was essential for gland development suggesting that it may be at the heart of the regulatory network controlling the gland-related genes expression. To investigate the relationship of *GhMYC2-like* and 6 *NACs*, RNAi transgenic plants were generated to interfere the transcript accumulation of *GhMYC2-like* in glanded cotton CCRI24. The gland development was repressed completely in all organs of RNAi plants (Figure S6). Expression analysis indicated that the transcript level of *GhMYC2-like* was suppressed extremely. Similarly, other 6 *NACs* were also down-regulated dramatically in RNAi plants suggesting that they functioned downstream of *GhMYC2-like*. In addition, unsurprisingly, transcript accumulation of *Gh_NAC120* was not interrupted (Figure 6 and Figure S7).

**Figure 7:**
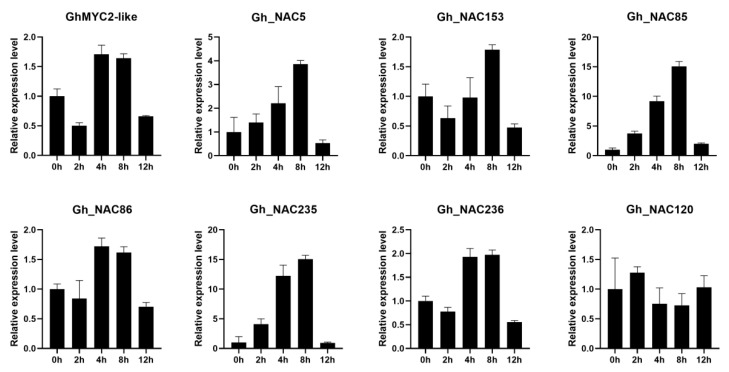
Dynamic expression analysis of *GhMYC2-like* and 7 *NAC* genes after MeJA treatment by qRT-PCR (reference gene: actin).

In previous reports, it was reviewed that MYC2 acted as a master regulator in jasmonate (JA) signaling pathway in Arabidopsis [57]. To analyze whether JA signaling pathway also involved in gland development, expression change of *GhMYC2-like* and *6 NAC* genes were detected at different time in cotton seedling which were subjected to MeJA treatment. As a result, all *GhMYC2-like* and 6 *NAC* genes were induced by MeJA and expression level reached the peak at 4 h or 8 h after treatment (Figure 7 and Figure S8). Among of them, *Gh_NAC85* and *Gh_NAC235* were highest sensitive to MeJA treatment and showed about 15 times of expression level at peak than 0 h stage. These results suggested the 6 *NACs* functioned downstream of *GhMYC2-like* and JA signaling pathway might be involved in gland development.

### 2.5. Silencing Gland-Related NACs Inffluences Gland Development

**Figure 8:**
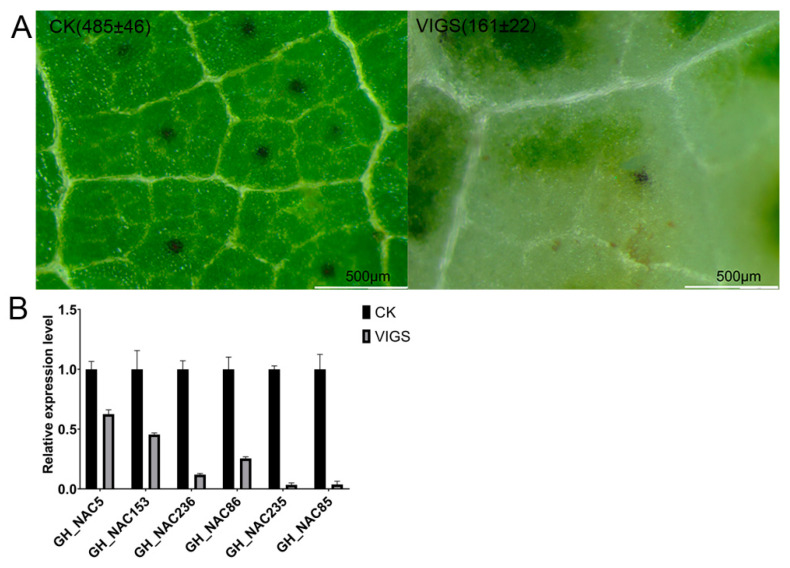
Silencing gland-related *NAC* genes causes reduction of gland number. (**A**) Microscopic images of VIGS plant leaf by stereomicroscope (Olympus SZX10). The number indicates gland number per cm^2^. (**B**) QRT-PCR analysis of genes expression in VIGS plants (reference gene: actin).

To check the function of gland-related *NACs*, virus induced gene silencing (VIGS) was performed to silencing the transcripts of the *NACs*. In previous report, silencing *CGF2*(*Gh_NAC5/Gh_NAC153*) reduced the number of gland by microscopic analysis [28]. In view of the high sequence similarity between *CGF2* and the other *4 NAC* genes (about 70%) and between the 4 *NACs* each other (more than 87%), the target sequence for VIGS was selected by sequence alignment (Figure S9). A 257 bp fragment was selected with low similarity between *CGF2* and the other 4 *NACs* for silencing the *4 NAC* genes specifically. Besides, the phytoene desaturase (*PDS*) gene was silenced with the *NACs* as a phenotypic marker for VIGS. As shown in Figure 8, silencing the *4 NAC* genes reduced gland number greatly. Accompany with dramatic silencing of *4 NAC* genes, the expression of *Gh_NAC5/Gh_NAC153* was also suppressed appreciably, which might also contribute to the reduction of gland number (Figure 8 and Figure S10). These results suggested that the *NAC* genes may be involved in gland development with functional redundancy.

### 2.6. CGF1 Influences Gland Formation by Regulating GhMYC2-Like and GLAND-Related NACs

**Figure 9:**
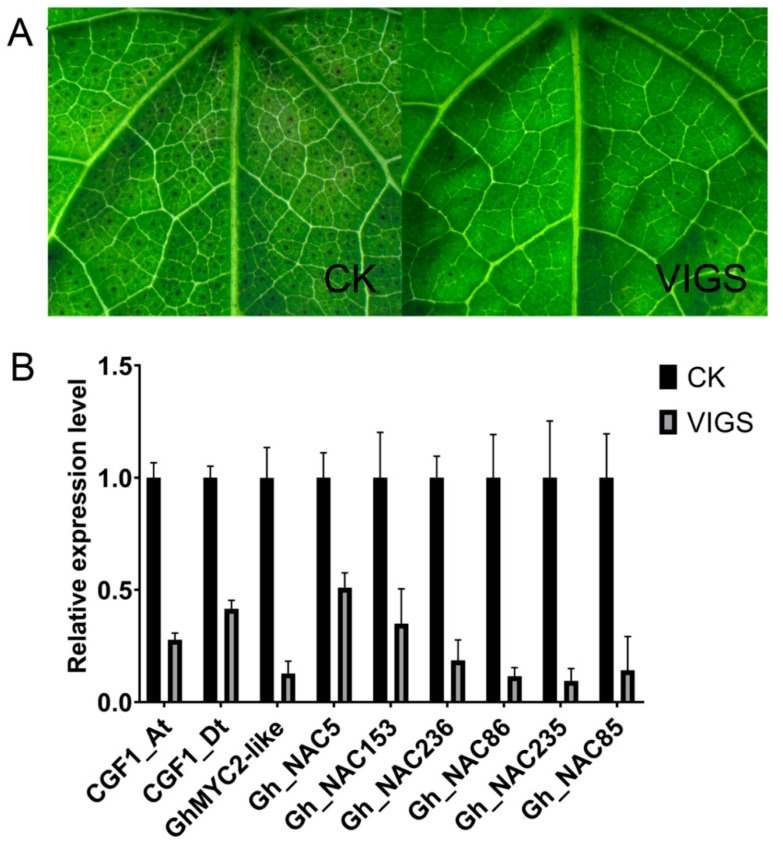
Silencing *CGF1* causes glandless phenotype and down-regulated expression of *GhMYC2-like* and *6 NAC* genes (reference gene: actin). (**A**) Phenotype of VIGS plants. (**B**) Expression analysis of *CGF1*, *GhMYC2-like* and *6 NAC* genes by qRT-PCR. CGF1_At: *CGF1* from A subgenome, CGF1_Dt: *CGF1* from D subgenome.

In the previous report, another MYC transcription factor CGF1 could influence gland formation significantly [28]. To explore how it functioned in gland formation, virus induced gene silencing (VIGS) was employed to knockdown *CGF1* expression in glanded cotton CCRI12. As shown in Figure 9, the gland number decreased observably in VIGS plants compared with control plants indicating important roles of *CGF1* in gland development (Figure 9A). Along with suppressed expression of *CGF1*, *GhMYC2-like* was also down-regulated dramatically (Figure 9B and Figure S11). Besides, the expression of *CGF1* was not influenced in RNAi-*GhMYC2-like* plants, indicating that *CGF1* functioned upstream of *GhMYC2-like* (Figure S11). Therefore, it is reasonable to conclude that *CGF1* participated in gland development process by regulating *GhMYC2-like*. In addition, as expected, the expression of 6 gland-related *NAC* genes was also reduced remarkably in VIGS-*CGF1* plants (Figure 9B and Figure S12). To check if *CGF1* influenced specifically gland-related *NAC* genes, other 9 *NACs* close to 6 gland-related *NACs* in phylogenetic tree were selected for qRT-PCR analysis. As shown in Figure S13, expression of eight genes was unaffected in VIGS-*CGF1* plants, except for one (*Gh_NAC87*) with a little reduced expression. We speculated that *CGF1* might specifically affect gland-related *NACs* expression by regulating *GhMYC2-like*.

### 2.7. Expression Profiling of 6 Gland-Related NAC Genes under Various Stresses

**Figure 10:**
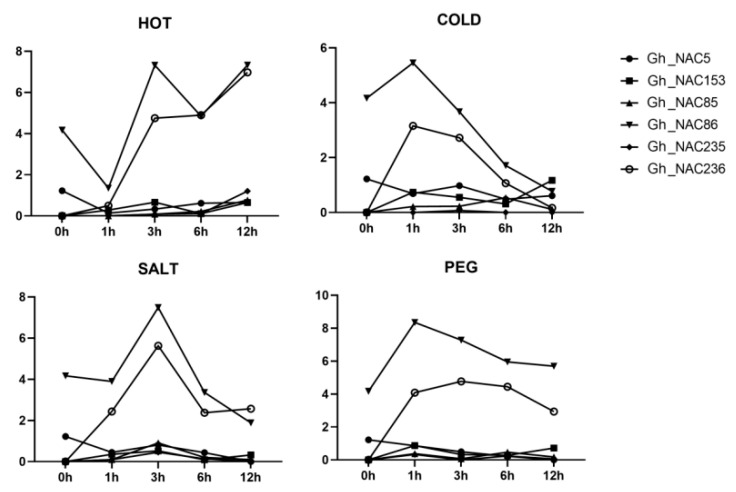
Expression changes of *6 NAC* genes after different stress treatments.

In previous studies, NAC family members were reported to be relative to stress responses [42]. To explore whether these *6 NAC* genes also have relation to stress response, dynamic expression of them were obtained from public RNA-seq data after various treatments, including cold, hot, salinity and polyethylene glycol (PEG). It can be seen in Figure 10, *Gh_NAC5*, *Gh_NAC85*, *Gh_NAC153* and *Gh_NAC235* were not sensitive to all treatments. *Gh_NAC86* and *Gh_NAC236* could response to the stress treatments and *Gh_NAC236* was more sensitive. Among of the treatments, hot and cold stresses caused opposite expression pattern of the genes. Along with the increase of the cold treatment time, expression of *Gh_NAC86* and *Gh_NAC236* were induced at 1 h and dramatic decline until 12 h back to initial state. On the contrast, they were still up-regulated extremely at 12 h after hot treatment. These results suggested that *Gh_NAC86* and *Gh_NAC236* also play roles in abiotic stress responses.

### 2.8. Predicted Regulatory Mechanism of the 6 NAC Genes

**Figure 11:**
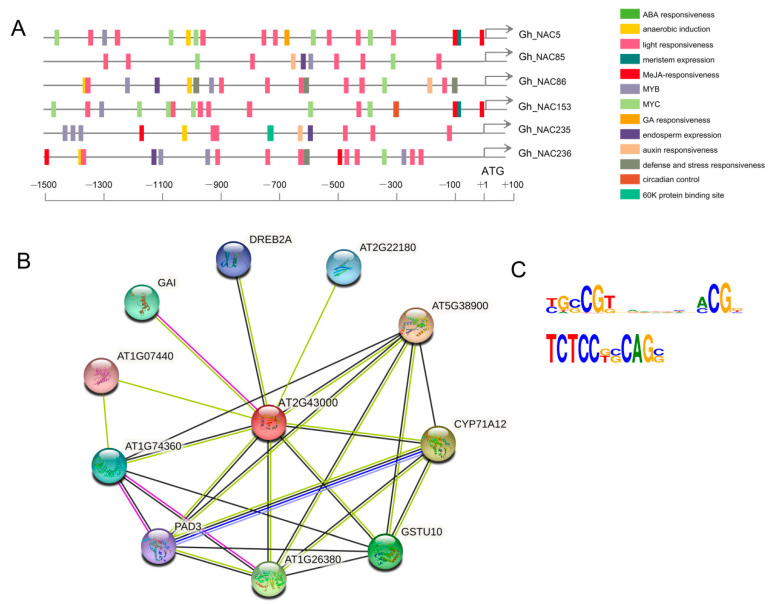
Predicted regulatory mechanism of *6 NAC* genes. (**A**) Cis-elements analysis of 6 *NAC* gene promoters. (**B**) Predicted interaction network of 6 NAC proteins. (**C**) Putative binding motifs of 6 NAC proteins.

To explore possible regulatory mechanism of the *6 NAC* genes, analysis of cis-elements and transcription factor binding sites (TFBS) were conducted in the 1500 bp promoter regions upstream of initiation codon. The results showed, except for common elements of CAAT-box and TATA-box, many cis-elements involved in light response were identified in all 6 *NAC* promoters. Plant hormone response elements were observed in different *NAC* promoters, including Methyl jasmonate (MeJA) response elements in *Gh_NAC5*, *Gh_NAC153*, *Gh_NAC235* and *Gh_NAC236*, auxin response elements in *Gh_NAC85*, *Gh_NAC86*, *Gh_NAC235* and *Gh_NAC236*, absence acid (ABA) response elements in *Gh_NAC5*, *Gh_NAC85*, *Gh_NAC153* and *Gh_NAC235*. In addition, unsurprising, defense and stress response elements were found only in promoter regions of *Gh_NAC86* and *Gh_NAC236*, whose expression was influenced by stress treatments (Figure 11A).

TFBS analysis showed that there were 33 transcription factors of 20 families which were predicted to bind to the promoter regions of 5 *NAC* genes except for *Gh_NAC85* (Figure S14 and Table S8). The TFs included members of Dof, GATA, HD-ZIP, MIKC_MADS, TCP, bHLH, MYB, ERF and C2H2, which were related to various development regulation and environmental stimuli. The CGF1 and GhMYC2-like were not included in the 33 transcription factors, which indicating that they probably indirectly regulated expression of the *NACs* through other factors.

Many evidences showed that miRNAs play important roles in plant development regulation [58]. To check whether miRNA also participated in gland development regulation, these 6 *NAC* gene sequences were used to scan potential miRNA targets in online software psRNATarget with all upland cotton miRNA sequences [59]. The result revealed that *Gh_NAC5* and *Gh_NAC153* might be targeted by ghr-miR482a/b and ghr-miR7506, as well as *Gh_NAC85* and *Gh_NAC235* by ghr-miR156a/b/d (Figure S15, Table S9).

To predict the interaction network of 6 NACs, the orthologs of each NAC were identified by using blastp search in protein database of The Arabidopsis Information Resource (TAIR) according to the score and E-value. The best match one was consider as the ortholog of each NAC and the 6 NACs share one common ortholog (AT2G43000) in Arabidopsis. AT2G43000 was used to screen the STRING database for interaction network prediction. As shown in Figure 11B, the NAC protein was likely to interact with different proteins to participate in different biological processes, such as biotic stress responses (CYP71A12, PAD3, AT1G26380 and AT5G38900), abiotic stress responses (GAI, DREB2A and AT1G07440) and signal transduction (AT1G74360). Besides, we conducted interaction network prediction of CGF1 and GhMYC2-like using their orthologs(AT5G46830 and AT4G17880)in STRING, respectively. One interaction network including CGF1 and GhMYC2-like could be constructed which mainly consist of members of JA signalling pathway, such as JAZs, COI and NINJA (Figure S16). This was consistent with the results above that JA signalling pathway could play important roles in pigment gland development. But, there was no overlap between the MYC-included and NAC-included interaction networks. These results suggested that the *6 NAC* genes might be involved in light and phytohormone signaling pathways and various biological processes regulated by different transcription factors or miRNAs. In addition, we predicted the binding sites of these 6 NAC proteins in footprintDB database by using protein sequence search in Arabidopsis database. The result showed that the NACs might bind two putative binding motifs to regulate downstream genes (Figure 11C).

## 3. Discussion

### 3.1. Identification and Phylogenetic Analysis of NAC Genes in Cotton

Plant-specific NAC protein family is one of the biggest transcription factor families, whose members participate in various stress responses and development regulation including PCD-associated development [47]. Although *NAC* genes have been identified in cotton [37,38,39,40,41], there is no report of integrated analysis of NAC members related to PCD-associated pigment gland development in cotton. With the development of new generation of sequencing technology, more accurate genomic information has been obtained, which lays a foundation for more accurate identification and analysis of gene functions [60,61]. In this study, we identified 150, 153 and 299 *NAC* genes in *G. arboreum*, *G. raimondii* and *G. hirsutum*. The numbers are larger than that reported before, which may be due to the better genome assembly [37,38]. Analysis of protein feature reveals that all NAC proteins are hydrophilic and most of them are predicted to be located in nucleus which conforms to the characteristics of transcription factors. However, unintelligibly, some NAC transcription factors are putative secreted proteins (extracellular proteins), which presumably do not act as transcription factors.

The phylogenetic tree shows that NAC proteins are divided into 8 groups in three cotton species. Almost all Ga_NACs and Gr_NACs appear with corresponding Gh_NACs in the phylogenetic tree branches, indicating their high conservation in 3 cotton genomes. But, some exceptions reflect the evolution divergence of NACs [38]. Gene duplication is the main force of gene family expansion [62]. In three cotton genomes, both segmental and tandem *NAC* gene duplications are identified contributing to family expansion (Table S5). Selective pressure analysis shows almost all *NAC* gene duplications are under purifying selection which consistent with previous reports [38,39]. Synteny analysis shows that plenty of *NAC* genes in cotton have corresponding orthologs in grape, Arabidopsis and cacao. These genes are relatively conserved during plants evolution implying their important roles in plant growth. Some *NAC* genes are only generated or retained in cotton suggesting that they are responsible for the unique characteristics of cotton, such as *Ga_NAC2/Gh_NAC2*, *Gr_NAC12/Gh_NAC151*, *Ga_NAC79/Gh_NAC81*, *Gr_NAC37/Gh_NAC231*, etc.

### 3.2. Expression Analysis and Potential Roles of NAC Genes in Pigment Gland Development

**Figure 12:**
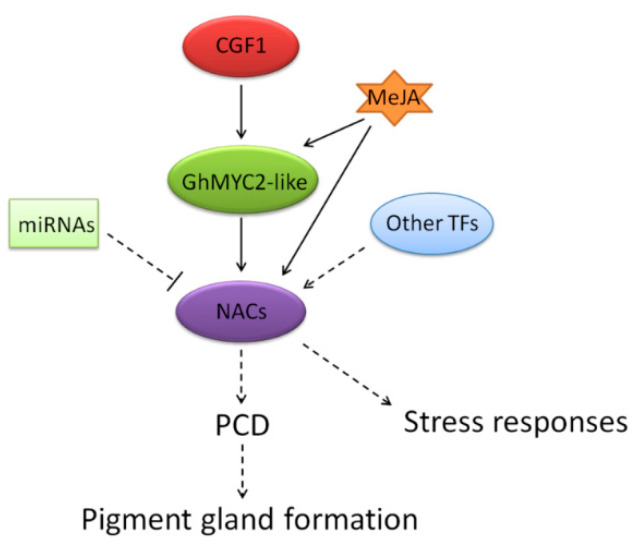
Putative MYC-NAC regulatory network in pigment gland formation.

Pigment glands are the main storage of gossypol and gland number has a close relation with gossypol content [16,17,18]. It is of great meaning to study gland-related genes for low-gossypol cotton breeding. In previous studies, GhMYC2-like (synonym GoPGF or CGF3) was confirmed to be the vital regulator of pigment gland development [26,27,28]. As a transcription factor, GhMYC2-like works by regulating other genes, but the regulatory network is still unclear now. Here, we conduct expression correlation analysis using *GhMYC2-like* gene as bait to screen co-expressed *NAC* genes using cotton RNA-seq data. Seven *NAC* genes are screened out with pearson correlation coefficient >0.5. It is notable that these 7 *NAC* genes are at low level expression before gland formation and turn into high level when gland development is starting in ovules. Then, it is further verified that 6 *NAC* genes among of them are low-expressed in all glandless materials suggesting they are involved in gland development. Meanwhile, Knockdown of *GhMYC2-like* causes extremely down-regulated expression of these 6 *NAC* genes, indicating they act downstream of *GhMYC2-like*. In the previous study, it is reported that knockout of *CGF2* (*Gh_NAC5/Gh_NAC153*) could reduce gland density but not stop gland formation [28]. In this study, we selected a small fragment with low similarity between *CGF2* and the other 4 *NAC* genes to silencing the latter specifically. Silencing the 4 *NAC* genes could also lead to reduction of gland number greatly. But, the expression of *CGF2* was also supressed appreciably, which might contribute to the low gland density as well. Therefore, we speculate that other 4 *NAC* genes (two pairs of tandem duplicated genes) may act together with *Gh_NAC5/Gh_NAC153* with functional redundancy in gland development and respective effect is weak. In addition, Silencing *CGF1* (another MYC transcription factor) could cause down-regulated expression of *GhMYC2-like* and *NACs*, leading to dramatic decreased gland number. It can be concluded that CGF1 acts as a regulator of *GhMYC2-like* to participate in gland development process. These results delineate a primary MYC-NAC transcription factors regulatory network in gland development in which GhMYC2-like acts as a key factor and downstream *NAC* genes or other factors lead to PCD to generate gland finally (Figure 12).

### 3.3. Putative Molecular Regulatory Mechanisms of Gland-Related NAC Genes

Phytohormone JA and its derivatives are key regulators in biotic stress responses [63]. In this study, *GhMYC2-like* and 6 *NAC* genes are induced by MeJA, indicating JA signaling pathway may be associated to gland development and the genes are likely to be involved in biotic stress responses. Perhaps they also contribute to the resistance of glanded cotton to some insects and pathogens. *Gh_NAC86* and *Gh_NAC236* can be also induced by abiotic stresses suggesting their potential function in abiotic stress responses (Figure 10). *Gh_NAC85* and *Gh_NAC235* are duplicated genes of *Gh_NAC86* and *Gh_NAC236* respectively, but they do not response to abiotic stresses, possibly due to lack of defense and stress response elements. This suggests the gene functional divergence may not only derive from coding sequence changes but also regulation region changes. Predicted regulatory mechanism of 6 *NAC* genes indicates that they might be regulated by light, various phytohormones and transcription factors, as well as miRNAs (Figure 12). Interaction network prediction suggests the 6 NAC proteins possibly enrich its functions by interacting with different proteins. Although need further confirmation, it implies their rich functionality and complex regulation network.

## 4. Materials and Methods

### 4.1. Identification and Phylogenetic Analysis of NAC Proteins

Newly published genome and protein sequences of *G. hirsutum* L. (ZJU version) [64], *G. arboreum* L. (CRI version) [65] and *G. raimondii* Ulbrich (JGI version) [66] were downloaded from CottonGen (https://www.cottongen.org, accessed on 20 June 2020). Genome and protein sequences of *Arabidopsis*, *Theobroma cacao* and *Vitis vinifera* were downloaded from EnsemblGenomes database (http://plants.ensembl.org/index.html, accessed on 20 June 2020). To identify NAC proteins, BLASTP and the hidden Markov model (HMM) search program were employed to search all protein sequences using the conserved No Apical Mristem (NAM) domain as bait as described before [40]. The domains of candidate NAC proteins were confirmed by online tool PfamScan (https://www.ebi.ac.uk/Tools/pfa/pfamscan/, accessed on 20 June 2020) [67]. The information of NAC proteins were obtained from cottonFGD (https://cottonfgd.org/, accessed on 20 June 2020) [68]. Subcellular localization analysis was made using ProtComp tool (http://linux1.softberry.com/, accessed on 20 June 2020). All NAC protein sequences were aligned using MAFFT and clustered by FastTree and phylogenetic tree was constructed using maximum likelihood (ML) method on cottonFGD (https://cottonfgd.org/, accessed on 20 June 2020) [68]. The phylogenetic tree was visualized in EvolView program (https://www.evolgenius.info//evolview/, accessed on 20 June 2020) [69].

### 4.2. Gene Chromosomal Location, Duplication and Synteny Analysis of NACs in Cotton

Chromosomal location of *NAC* genes were determined based on the genome annotation information and illuminated with TBtools [70]. The gene duplication analysis of three cotton species were conducted using MCScanX software by criterion of sequence coverage and similarity of aligned sequence both more than 80% [71]. The synonymous substitution (Ks) and nonsynonymous substitution (Ka) of each duplicated gene pairs were calculated in TBtools with default parameters [70]. Synteny analysis was carried out using JCVI python package (https://github.com/tanghaibao/jcvi/, accessed on 20 June 2020) [72]. Gene duplication and synteny relationship were visualized by TBtools [70].

### 4.3. Gene Expression and Correlation Analysis of GhMYC2-Like and NAC Genes

RNA-seq data (accession: PRJNA248163) was downloaded from European Nucleotide Archive (ENA) database. The samples included different development stages of root and ovlue and various tissues of *G. hirsutum* TM-1. Salmon tool was employed to quantify the gene expression by Transcripts Per Million (TPM) [73]. All gene expression levels were normalized by log2 (TPM+1) and pearson correlation analysis was performed using R package psych. The heatmaps of genes expression were made with R package pheatmap.

### 4.4. Plant Materials and MeJA Treatment

To verify the *NAC* genes relative to gland phenotype, genes expression were analyzed by qRT-PCR in various cotton lines, including glanded cotton CCRI12, Liao7, CCRI17, Dominant glandless (Dgl) cotton Dgl-CCRI12 (with *Gl_2_^e^*), Dgl-Liao7 (with *Gl_2_^e^*) and Recessive glandless (Rgl) cotton Rgl-CCRI12 (with *gl_2_* and *gl_3_*), Rgl-CCRI17 (with *gl_2_* and *gl_3_*), as well as stem-glandless line T582 (with *gl_1_*). All the plant materials were from National Medium-term Gene Bank of Cotton (Anyang, China) and maintained by selfing in our lab. RNAi transgenic plants were generated by agrobacterium-mediated genetic transformation to interfere accumulation of *GhMYC2-like* transcripts. For MeJA treatment, Liao7 plants grew in green house with 27 °C (16 h light/8 h dark) and the leaves at three-leaf stage were treated by 100 μM MeJA. The treated leaves were collected at 0 h, 2 h, 4 h, 8 h and 12 h after treatment, respectively.

### 4.5. Virus Induced Gene Silencing (VIGS)

A 628-bp specific fragment of *CGF1* gene was amplified and inserted to pTRV2, a tobacco rattle virus vector. For VIGS of the *NAC* genes and *PDS* gene simultaneously, the fragments of two genes were inserted to pTRV2 in tendam. Silencing *PDS* gene could lead to plant bleaching, which was used as phenotypic marker for VIGS. The pTRV2-*CGF1*, pTRV2 and pTRV1 were transferred into *Agrobacterium tumefaciens* GV3101, respectively. The CCRI12 plants grew in illumination incubator with 16 h/8 h light/dark at 25 °C. The VIGS procedure was performed according to the method reported before [74]. Three weeks later, the gland phenotype was recorded and leaves of VIGS plants were collected for RNA extraction and qRT-PCR.

### 4.6. RNA Extraction and QRT-PCR Analysis

The stem or leaf tissues of the plant materials were collected and put in liquid nitrogen immediately. Total RNA was extracted using RNAprep Pure Plant Kit (Tiangen, Beijing, China) according to the instructions. The first cDNA strand synthesis was made using PrimeScript II 1st Strand cDNA Synthesis Kit (TAKARA, Dalian, China). The qRT-PCRs were performed on ABI QuantStudio5 RT-PCR system (Applied Biosystems, Foster City, CA, USA) with three replicates using TB Green *Premix Ex Taq* Kit (TAKARA, Dalian, China). PCR reaction conditions were as follows: 95 °C for 30 s; 40 cycles at 95 °C for 5 s and 60 °C for 30 s; followed by 95 °C for 15 s, 60 °C for 1 min, 95 °C for 15 s. ACTIN (Genbank id AY305733), ubiquitin (Genbank id EU604080) and histone (Genbank id AF024716) were employed as internal controls. Relative gene expression was calculated using the 2^−ΔΔCT^ method. All primers information was listed in Table S1.

### 4.7. Predictions of Cis-Elements, TFBS, miRNA Targets, Interaction Network and Binding Sites

The 1.5 kb promoter sequences of *NAC* genes were extracted upstream of initiation codon. All sequences were submitted to PlantCARE (http://bioinformatics.psb.ugent.be/webtools/plantcare/html/, accessed on 20 June 2020) to search putative cis-elements. TFBS predications were performed using Binding Site Prediction tool in PlantTFDB database (http://planttfdb.cbi.pku.edu.cn/, accessed on 20 June 2020) with *p* value < 1 × 10^−6^. For miRNA targets prediction, *NAC* genes sequences were submitted to psRNATarget server to check the possible target sites by miRNAs of upland cotton with default parameters. Because of the well-studied interaction network in Arabidopsis, interaction network prediction of the 6 NAC proteins were performed in STRING database (http://string-db.org/cgi/input.pl, accessed on 20 June 2020) using their orthologs in Arabidopsis. To obtain the putative binding sites, the 6 NAC proteins sequences were submitted to footprintDB database (http://floresta.eead.csic.es/footprintdb/index.php, accessed on 20 June 2020) to search putative binding sites.

## Data Availability

Not applicable.

## Supplementary Materials

The following are available online at https://www.mdpi.com/article/10.3390/ijms22095007/s1, Figure S1 Chromosomal location of cotton *NACs*. (A) and (B) Chromosomal location of *NACs* from A subgenome and D subgenome of *G. hirsutum*, respectively. (C) and (D) Chromosomal location of *NACs* from *G. arboreum* and *G. raimondii*, respectively. Numbers on the left indicate the length of chromosome. Black lines indicate the tandem duplication relationship. Ga: *Gossypium arboreum*, Gr: *Gossypium raimondii*, A/D: A/D subgenome of *Gossypium hirsutum*. The numbers indicate chromosome number. Figure S2 Synteny analysis of *NACs* in *G. hirsutum*. Red lines indicate the collinear relationship. Blue and purple blocks indicate chromosomes of A subgenome and D subgenome. A/D: A/D subgenome of *Gossypium hirsutum*. The numbers indicate chromosome number. Figure S3 Synteny analysis of *NACs* in *G. arboreum*. Red lines indicate the collinear relationship. Ga: *Gossypium arboreum*, the numbers indicate chromosome number. Figure S4 Synteny analysis of *NACs* in *G. raimondii*. Red lines indicate the collinear relationship. Gr: *Gossypium raimondii*. The numbers indicate chromosome number. Figure S5 Expression analysis of *GhMYC2-like* and *7 NAC* genes in glanded and glandless cotton. Reference gene: ubiquitin (A) and histone (B). CCRI: China Cotton Research Institute, Dgl: dominant glandless, Rgl: recessive glandless, L7: Liaomian7. Figure S6 Phenotype of RNAi plants interferencing the expression of *GhMYC2-like*. (A–C) indicate the glandless leaf, seed and hypocotyl of RNAi plants. (D–F) indicate the glanded leaf, seed and hypocotyl of CCRI24 plants. Figure S7 QRT-PCR analysis of *GhMYC2-like* and *7 NAC* genes in CCRI24 and RNAi plants. Reference gene: ubiquitin (A) and histone (B). Figure S8 Dynamic expression analysis of *GhMYC2-like* and *7 NAC* genes after MeJA treatment by qRT-PCR. Reference gene: ubiquitin (A) and histone (B). Figure S9 6 *NAC* gene sequences alignment. The red boxes indicate target region for VIGS. Figure S10 Silencing gland-related *NAC* genes causes reduction of gland number. (A) Phenotype of VIGS plants. (B)and(C) QRT-PCR analysis of genes expression in VIGS plants. Reference gene: ubiquitin (B) and histone (C). Figure S11 Expression analysis of *CGF1* in RNAi-*GhMYC2-like* plants. Reference gene: actin (A), ubiquitin (B) and histone (C). Figure S12 Silencing *CGF1* causes glandless phenotype and down-regulated expression of *GhMYC2-like* and *6 NAC* genes. Reference gene: ubiquitin (A) and histone (B). Figure S13 Expression analysis of *9 NAC* genes with close relation to the 6 gland-related *NAC* genes in *CGF1*-VIGS plants. Reference gene: actin (A), ubiquitin (B) and histone (C). Figure S14 TFBS analysis of 6 *NAC* gene promoters. The numbers indicate the distance (in base pairs) upstream (−) or downstream (+) of the translation initiation codon ATG. Figure S15 miRNA targets prediction of 6 *NAC* genes. Blue blocks indicate the NAM conserved domain. Red blocks indicate the target sits of miRNAs. Figure S16 Interaction network of GhMYC2-like and CGF1. Table S1 All primers information in this study. Table S2 *Gh_NAC* genes information. Table S3 *Ga_NAC* genes information. Table S4 *Gr_NAC* genes information. Table S5 *NAC* gene duplications information in three cotton genomes. Table S6 Orthologious *NAC* genes of three cotton genomes. Table S7 Orthologious *NAC* genes between cotton and A. thaliana, T. cacao and V. vinifera. Table S8 Predicted TFBS information. Table S9 Information of predicted miRNA targets of 6 *NAC* genes.
