# Supplementary figures and images for: Gene Expression Correlation Analysis Reveals MYC-NAC Regulatory Network in Cotton Pigment Gland Development
Source: Int J Mol Sci. 2021 May 8;22(9):5007. doi: 10.3390/ijms22095007 (PMC8125883; doi:10.3390/ijms22095007)

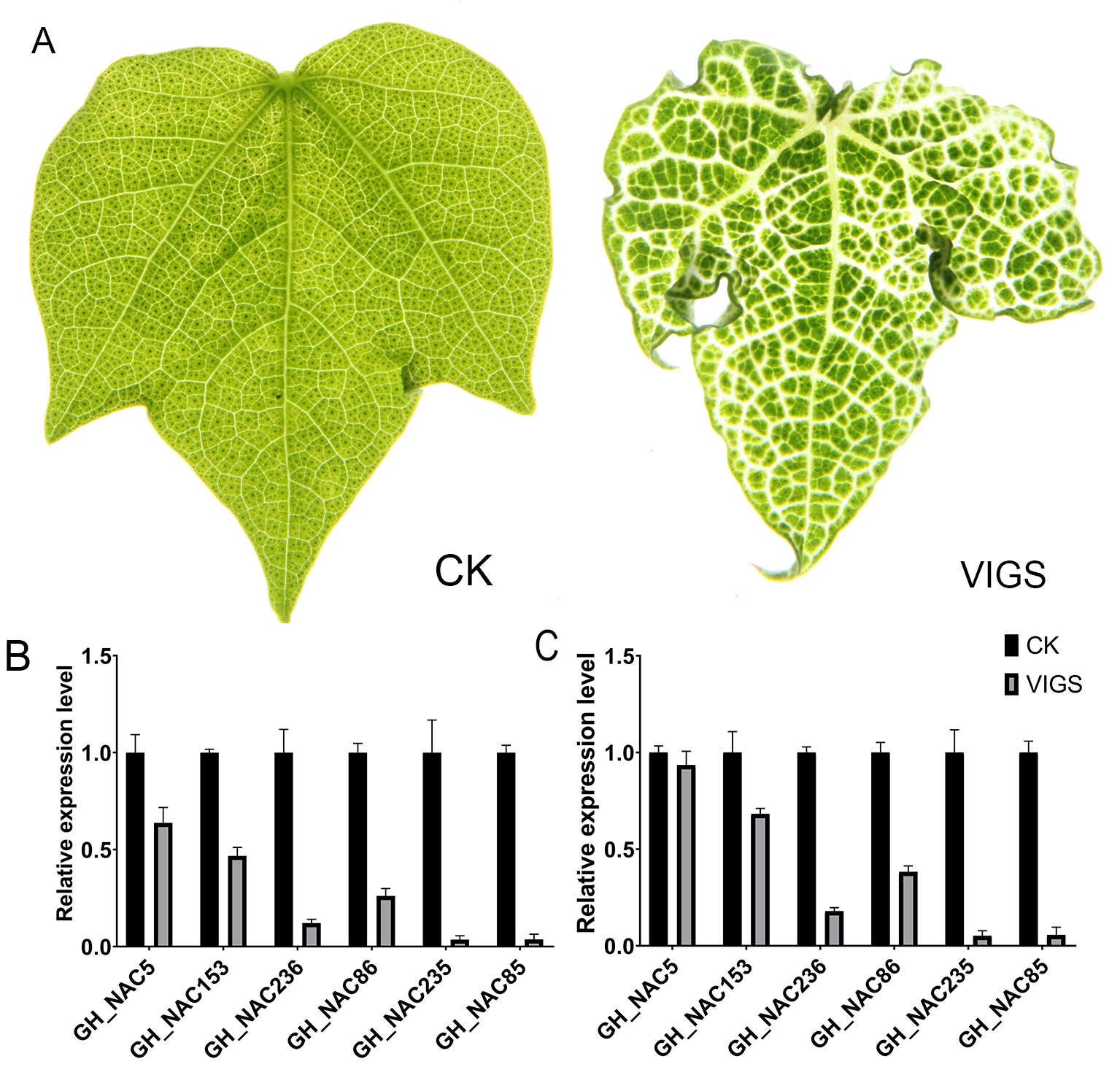

Supplement: Supplementary file 1 [file ijms-22-05007-s001.zip › FIGURE S10 Vigs NAC ubiq 21.4.21.tif]

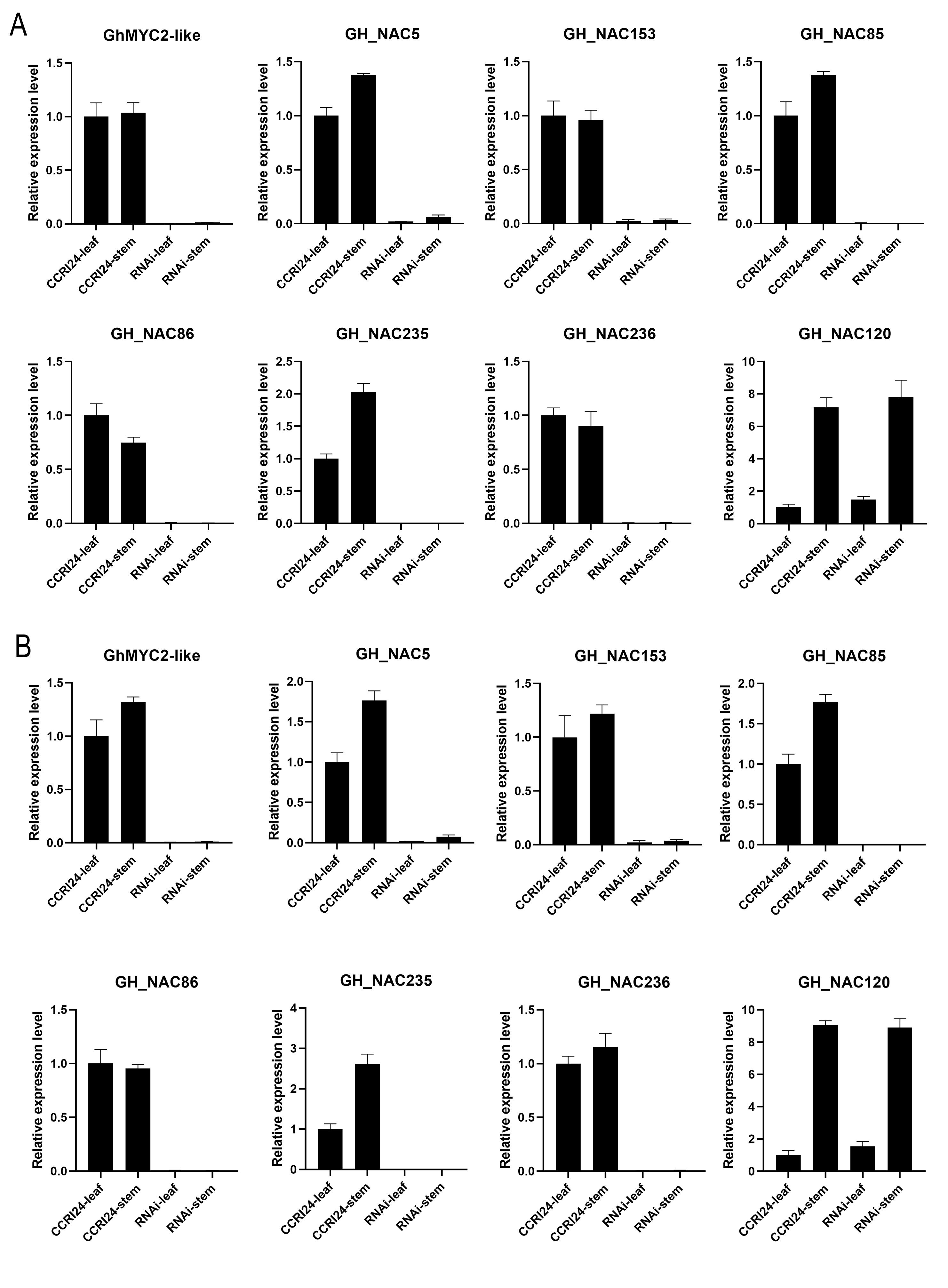

Supplement: Supplementary file 1 [file ijms-22-05007-s001.zip › FIGURE S7 RNAi ubiq NAC exp 21.4.21.tif]

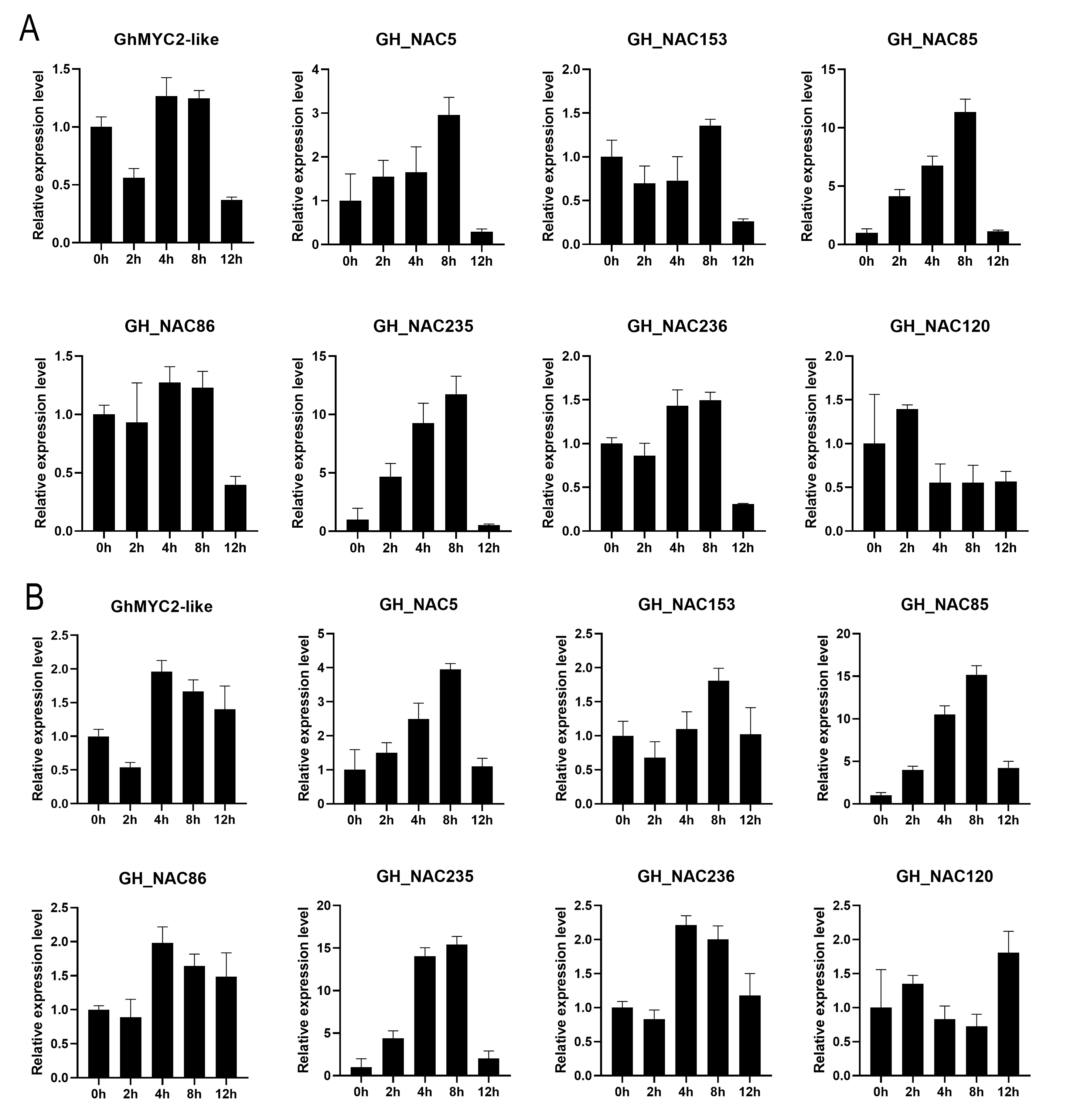

Supplement: Supplementary file 1 [file ijms-22-05007-s001.zip › FIGURE S8 JA ubiq merge 21.4.21.tif]

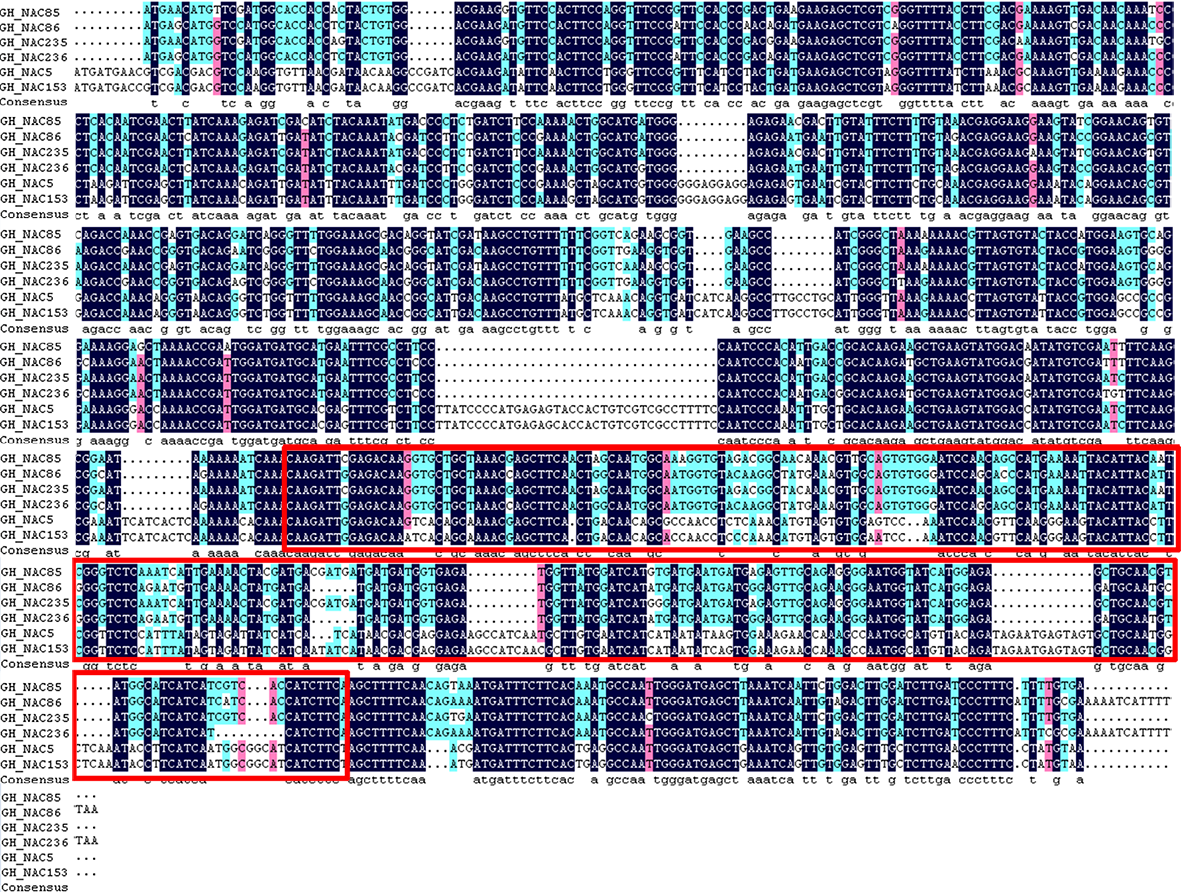

Supplement: Supplementary file 1 [file ijms-22-05007-s001.zip › FIGURE S9 SEQ alignment.tif]

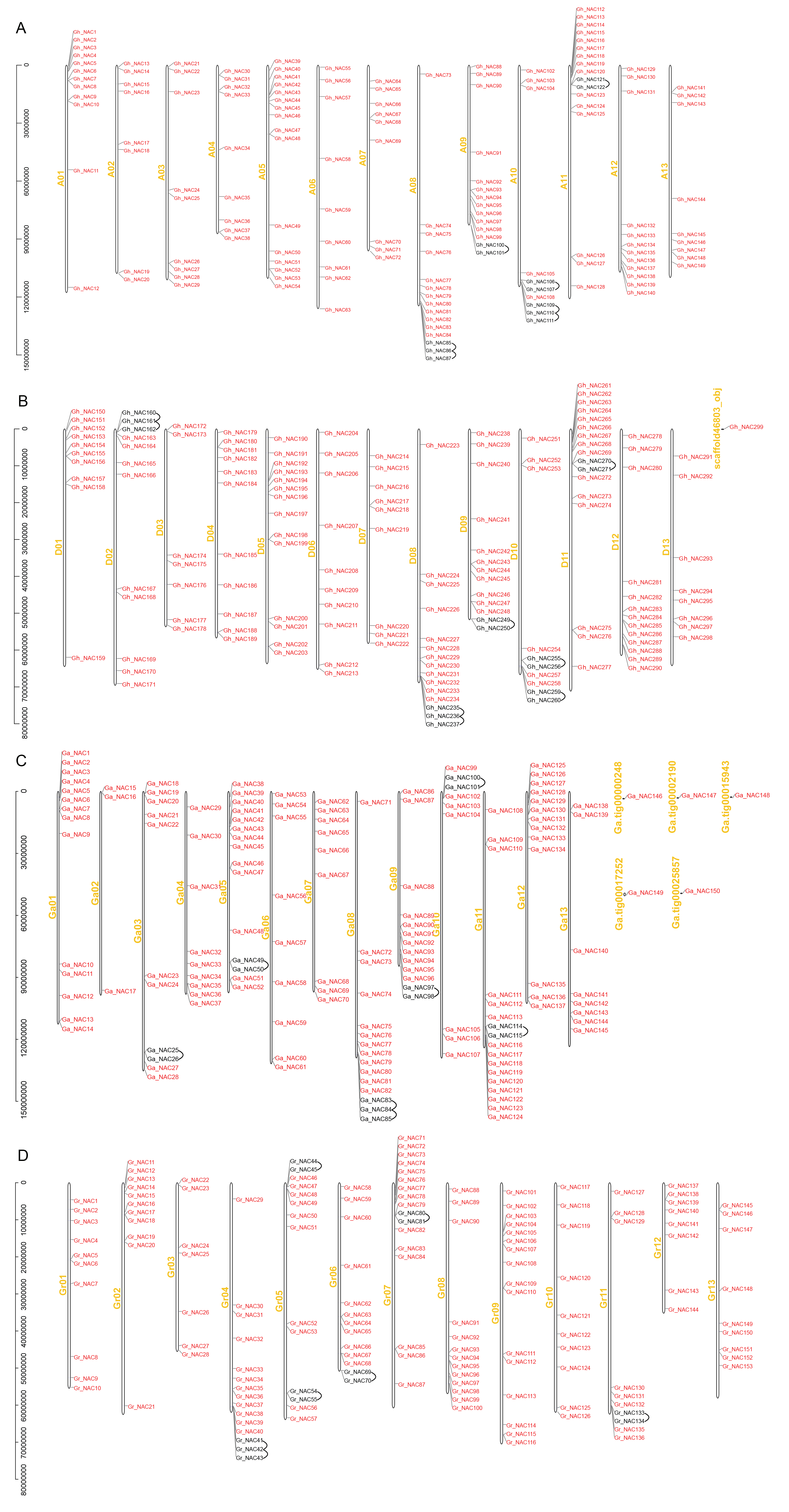

Supplement: Supplementary file 1 [file ijms-22-05007-s001.zip › Figure S1.tif]

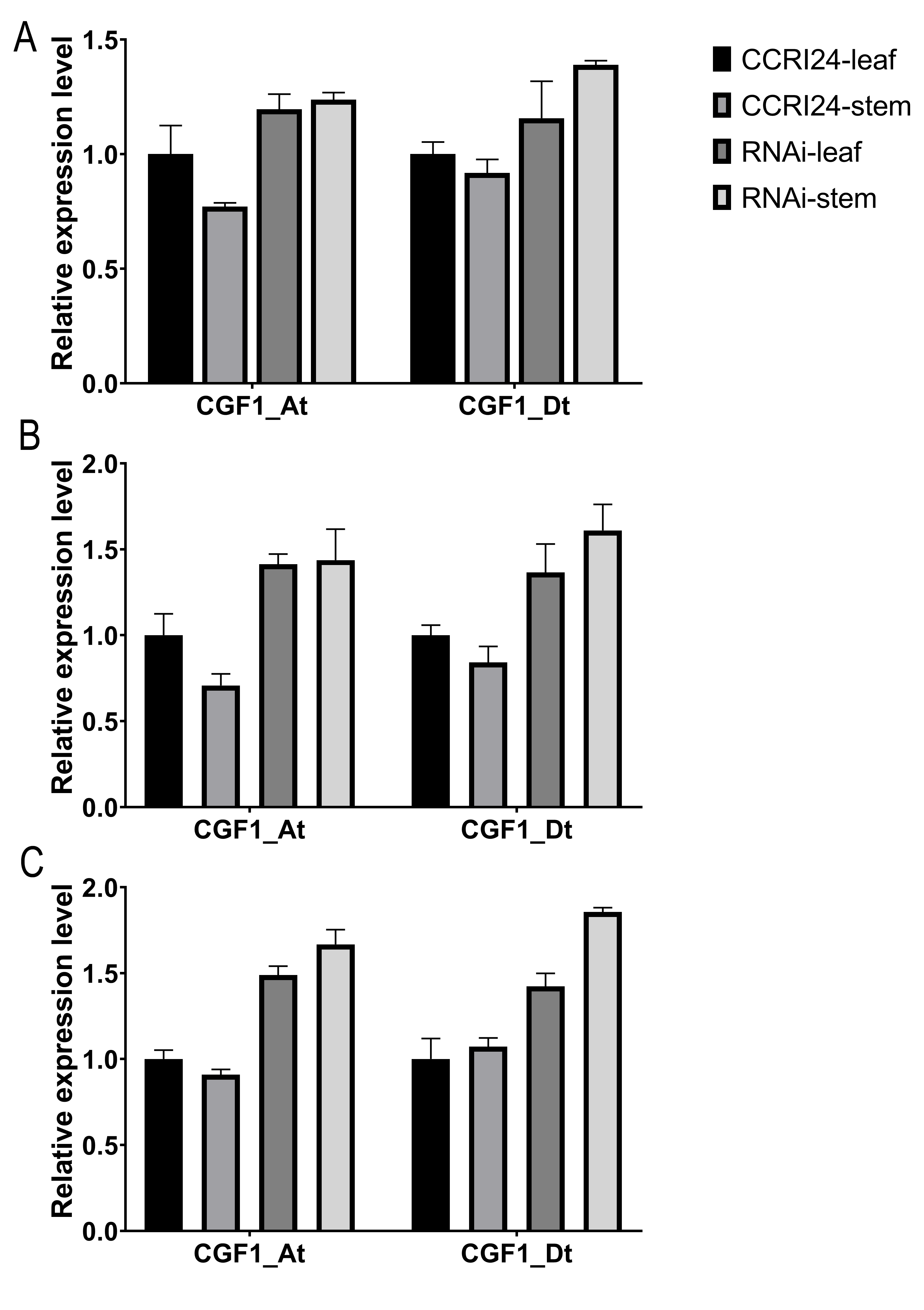

Supplement: Supplementary file 1 [file ijms-22-05007-s001.zip › Figure S11 2021.4.21.tif]

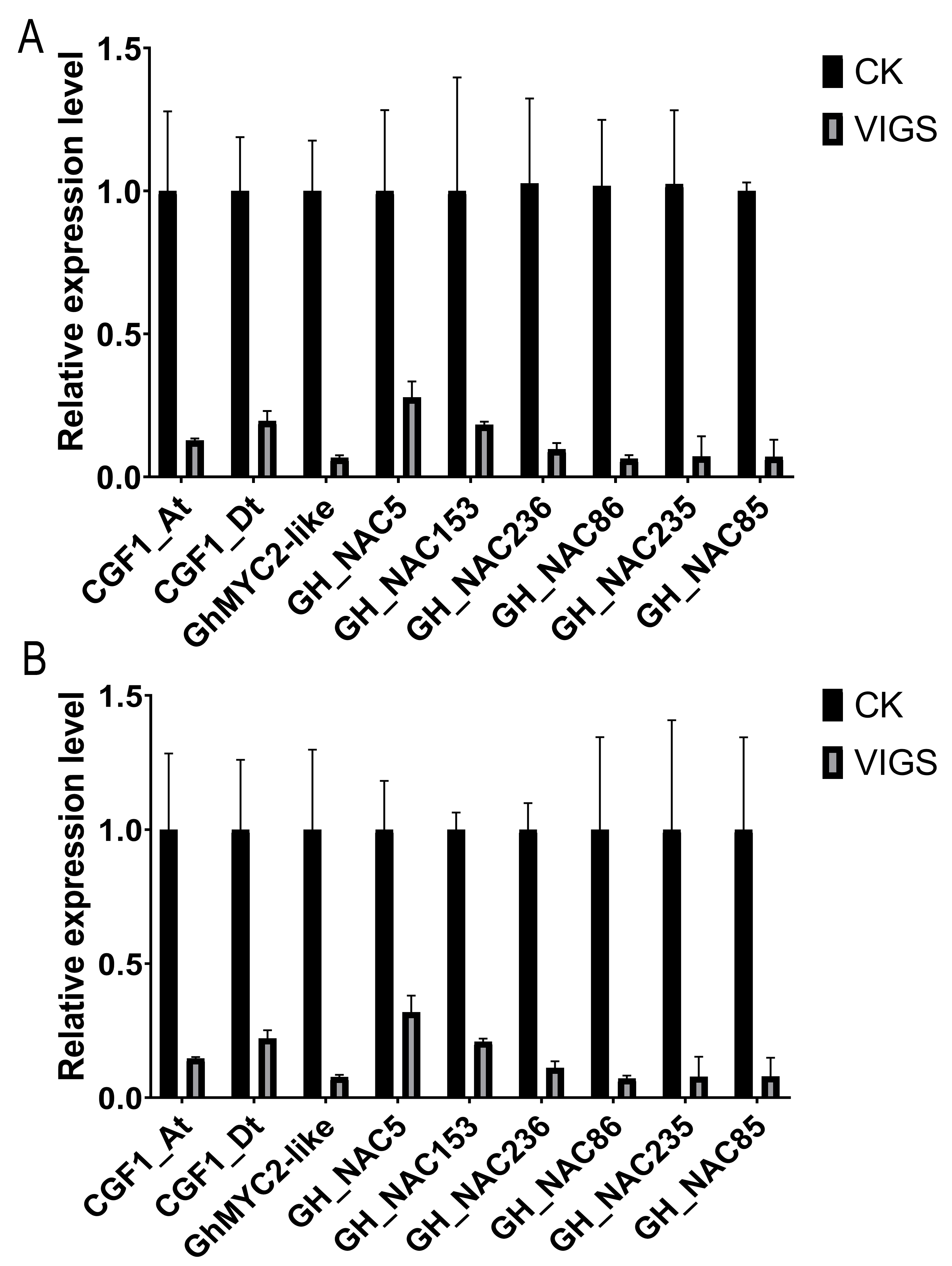

Supplement: Supplementary file 1 [file ijms-22-05007-s001.zip › Figure S12 OV1055 genes exp UBIQ HIS 21.4.21.tif]

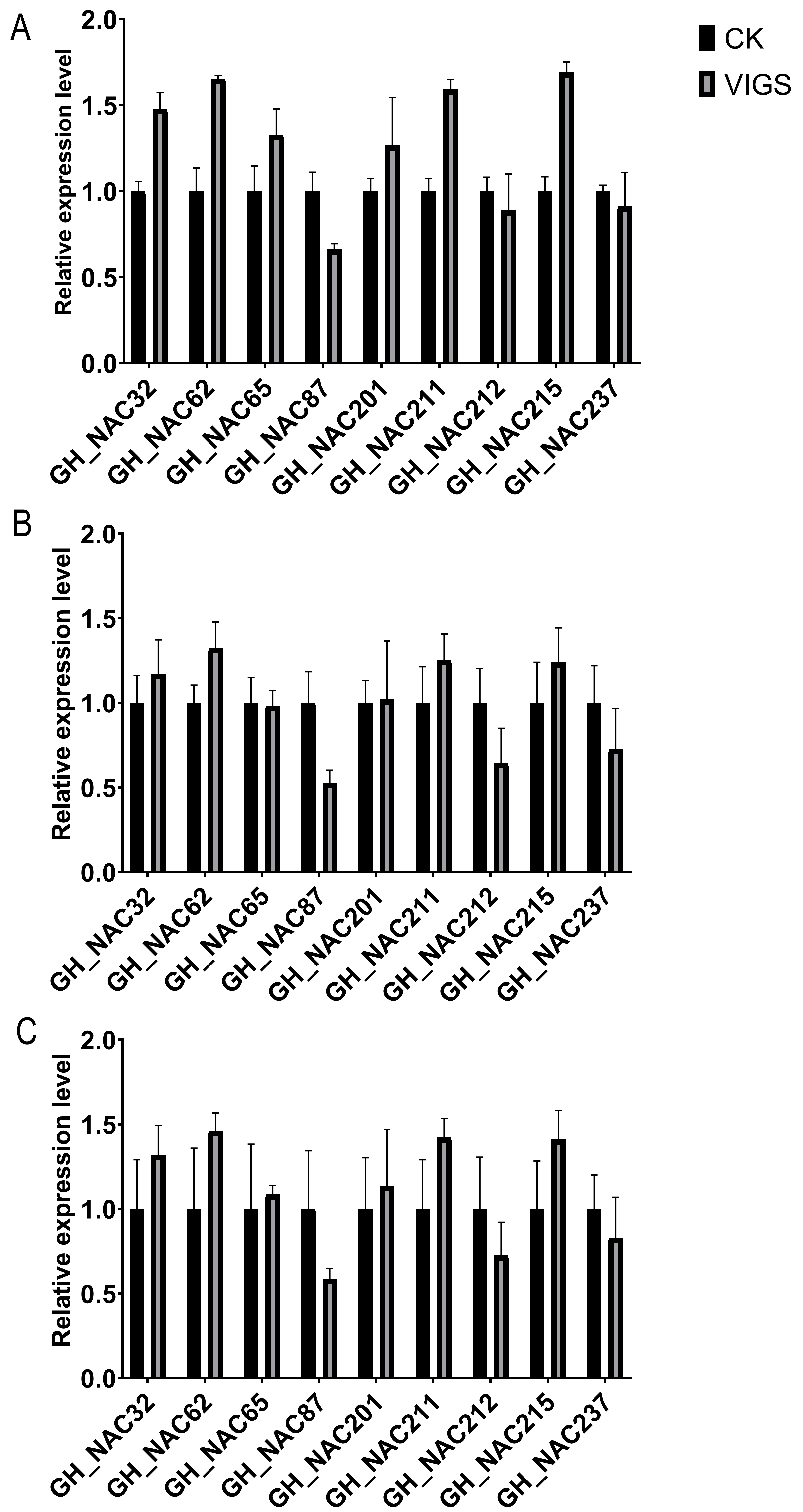

Supplement: Supplementary file 1 [file ijms-22-05007-s001.zip › Figure S13 2021.4.21.tif]

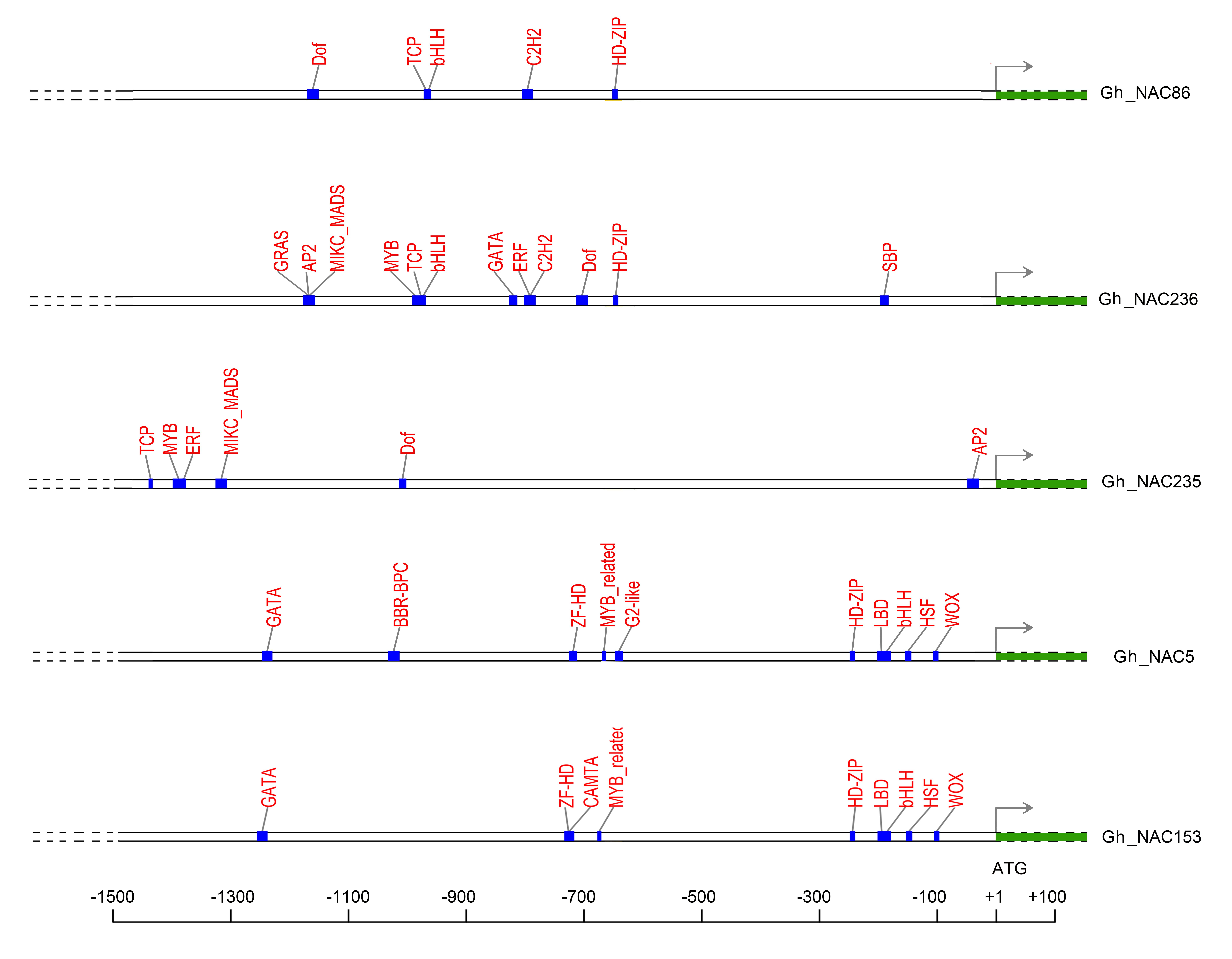

Supplement: Supplementary file 1 [file ijms-22-05007-s001.zip › Figure S14.tif]

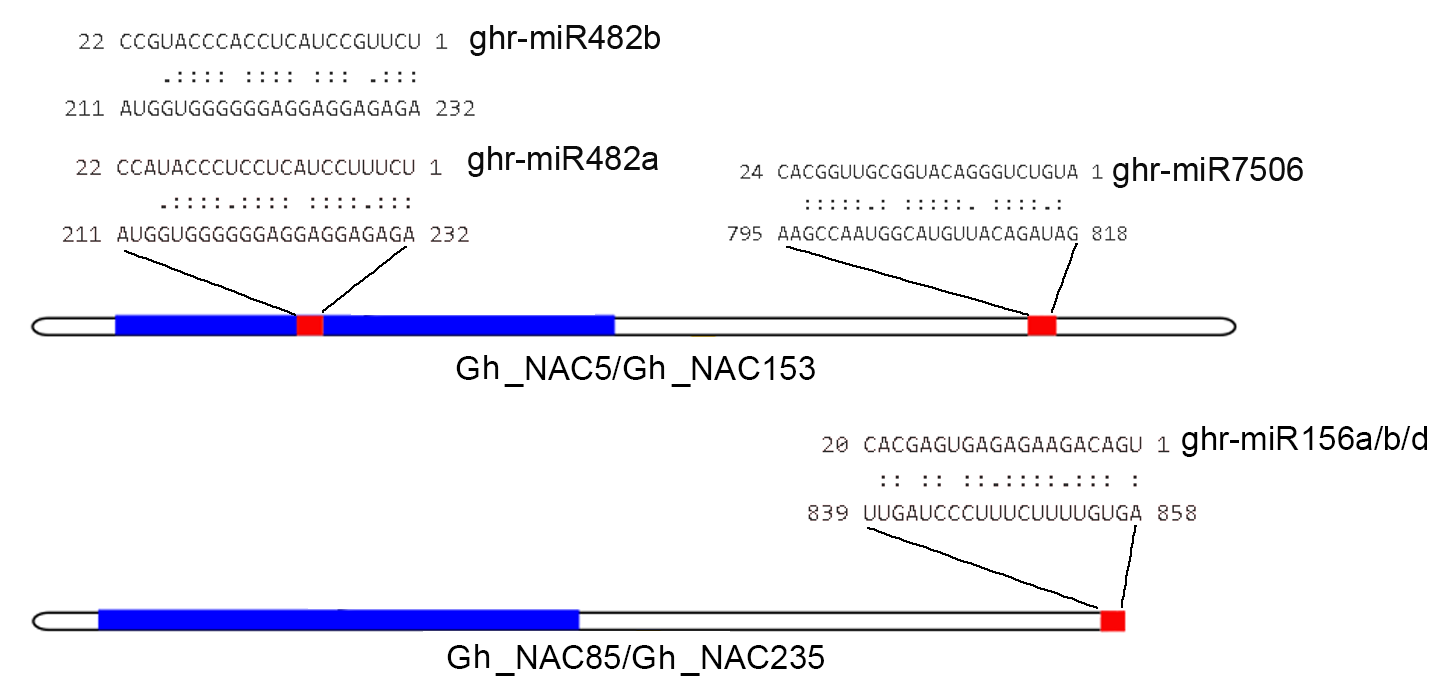

Supplement: Supplementary file 1 [file ijms-22-05007-s001.zip › Figure S15.tif]

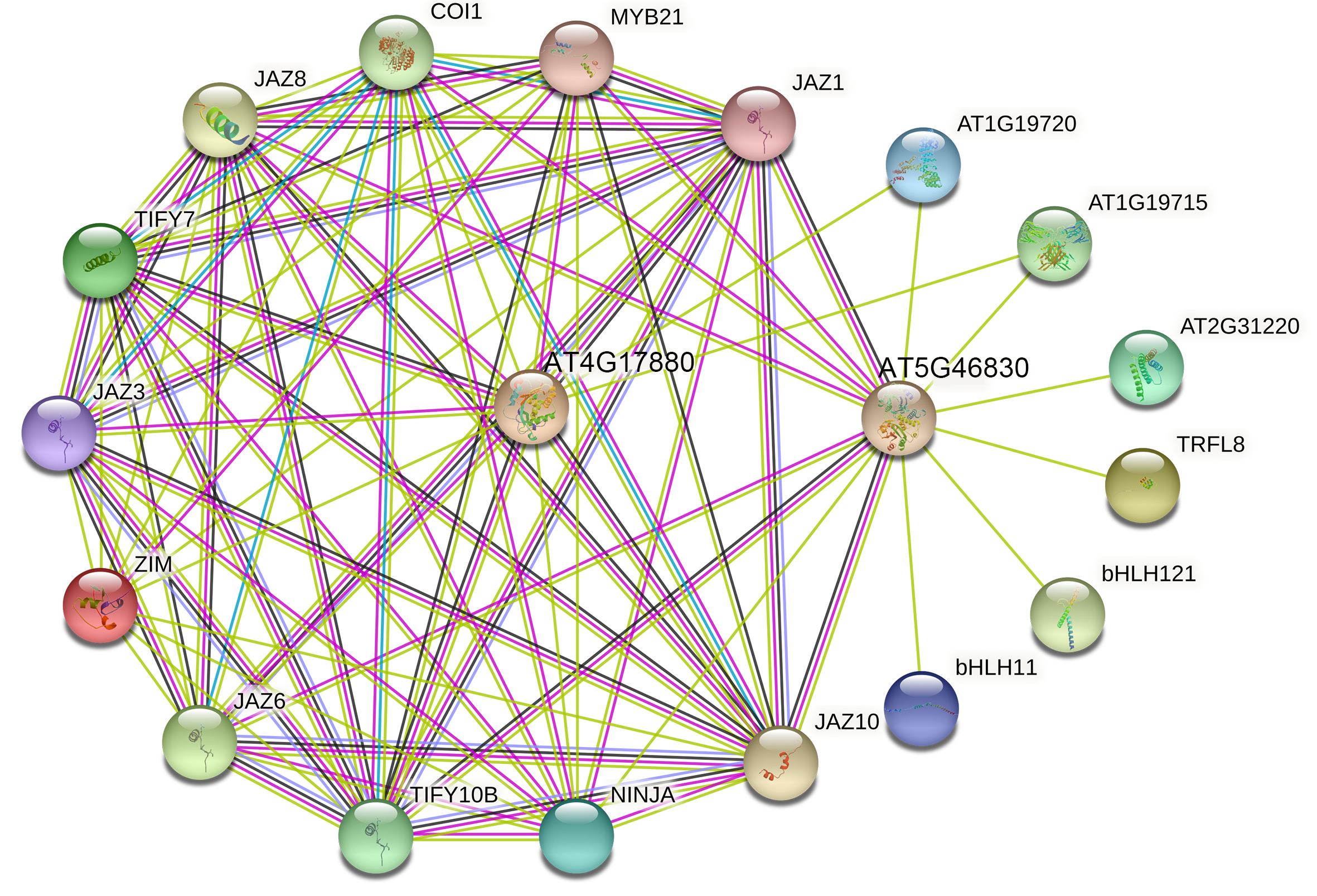

Supplement: Supplementary file 1 [file ijms-22-05007-s001.zip › Figure S16 2020.12.14.tif]

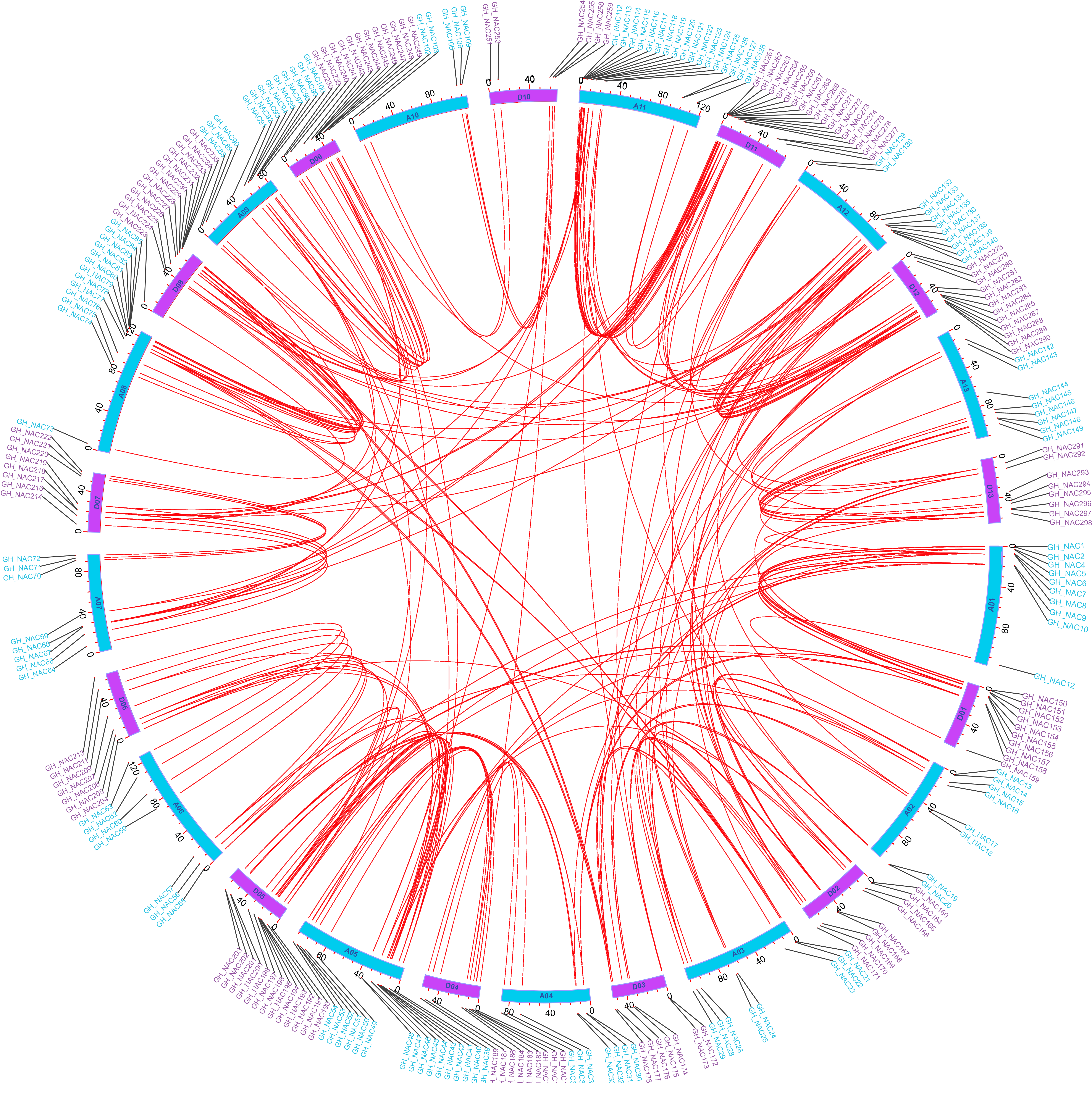

Supplement: Supplementary file 1 [file ijms-22-05007-s001.zip › Figure S2.tif]

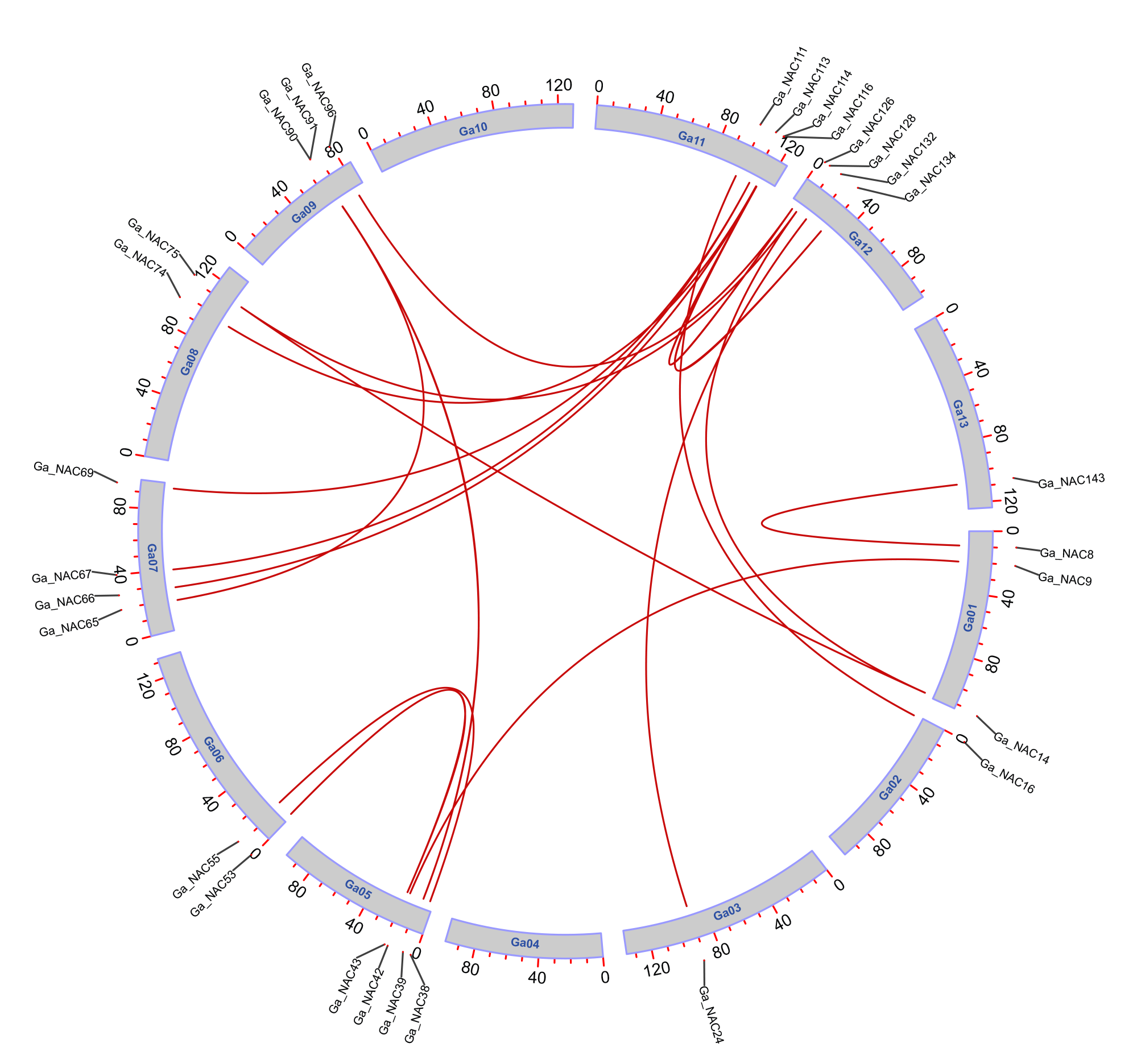

Supplement: Supplementary file 1 [file ijms-22-05007-s001.zip › Figure S3.tif]

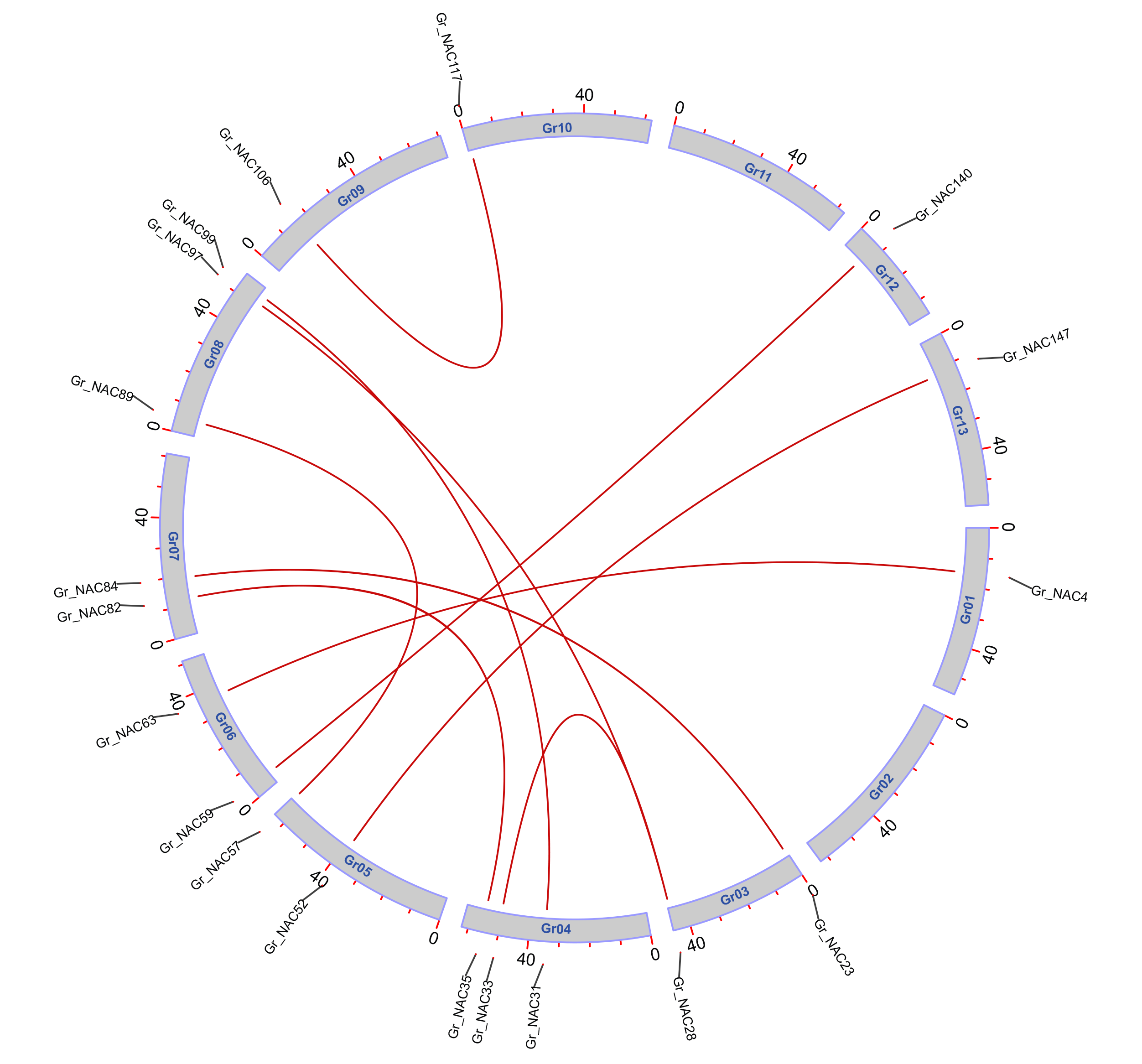

Supplement: Supplementary file 1 [file ijms-22-05007-s001.zip › Figure S4.tif]

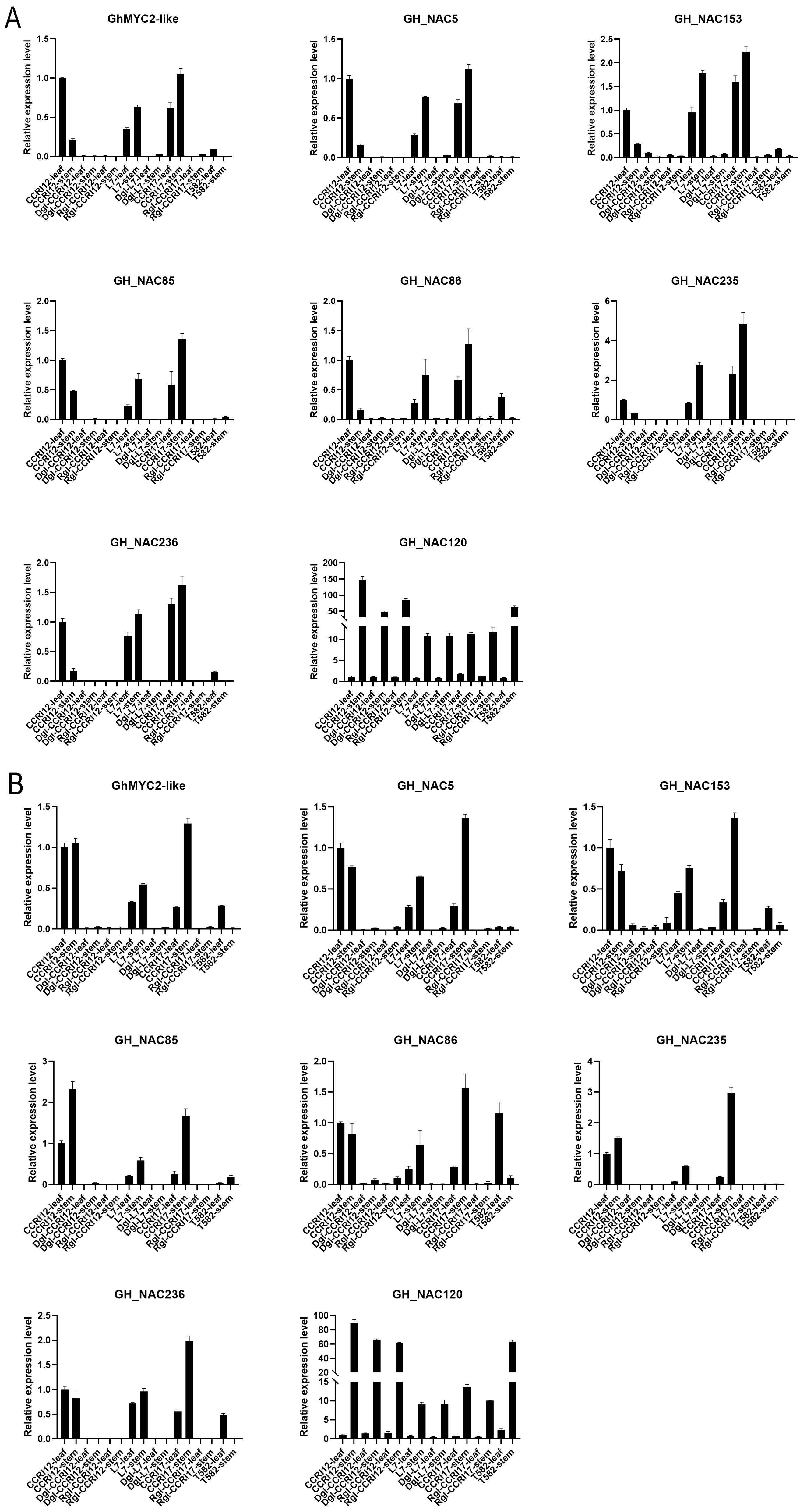

Supplement: Supplementary file 1 [file ijms-22-05007-s001.zip › Figure S5 2021.4.21.tif]

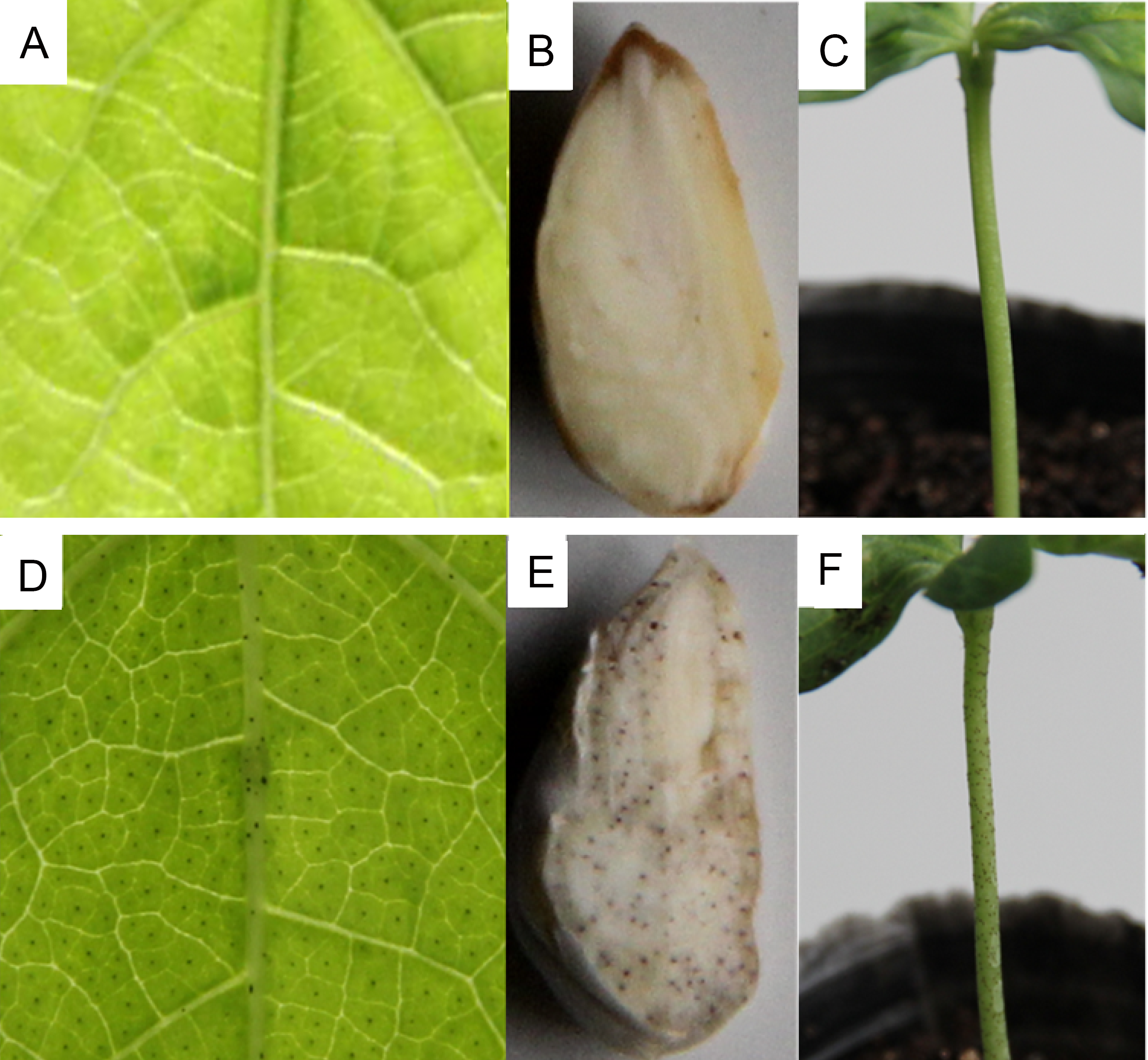

Supplement: Supplementary file 1 [file ijms-22-05007-s001.zip › Figure S6.tif]
